# Caloric Restriction and Sirtuins as New Players to Reshape Male Fertility

**DOI:** 10.3390/metabo15050303

**Published:** 2025-05-02

**Authors:** Diana C. A. André, Pedro F. Oliveira, Marco G. Alves, Ana D. Martins

**Affiliations:** 1 LAQV-REQUIMTE, Department of Chemistry, University of Aveiro, 3810-193 Aveiro, Portugal; dianacaa@ua.pt (D.C.A.A.); p.foliveira@ua.pt (P.F.O.); 2 Institute of Biomedicine, Department of Medical Sciences (iBiMED), University of Aveiro, 3810-193 Aveiro, Portugal; marcoalves@ua.pt

**Keywords:** caloric restriction, male fertility, protein (de)acetylation, sirtuins, spermatogenesis

## Abstract

Over the years, caloric intake has remained a subject of profound scrutiny. Within the scientific community, there has been rigorous debate to ascertain which path is most ideal for enhancing quality of life and extending the human lifespan. Caloric restriction has been shown to be a promising contributor towards longevity and delaying the onset of age-related diseases. This diet consists of a reduction in caloric intake while maintaining essential energy and nutritional requirements to achieve optimal health while avoiding malnutrition. However, the effects of this nutritional regimen on male reproductive health have not yet been comprehensively studied. Nevertheless, such a complex process will certainly be regulated by a variety of metabolic sensors, likely sirtuins. Evidence has been gathered regarding this group of enzymes, and their ability to regulate processes such as chromatin condensation, the cell cycle, insulin signaling, and glucose and lipid metabolism, among many others. Concerning testicular function and male fertility, sirtuins can modulate certain metabolic processes through their interaction with the hypothalamic–pituitary–gonadal axis and mitochondrial dynamics, among many others, which remain largely unexplored. This review explores the impact of caloric restriction on male fertility, highlighting the emerging role of sirtuins as key regulators of male reproductive health through their influence on cellular metabolism.

## 1. Introduction

Over the years, caloric intake has remained a subject of profound scrutiny and discussion. Insights on what to eat and how much to eat, and the prevailing aesthetic and cultural norms that influence these perceptions, have undergone a continuous evolution over recent decades. These shifts in dietary models have given rise to a diverse spectrum of nutritional regimens, each defined by unique dietary requirements and schedules. The scientific community has explored various dietary methodologies, engaging in rigorous debate to ascertain which approach offers the most objectively refined path towards enhancing the quality of life and extending the human lifespan.

**Figure 1:**
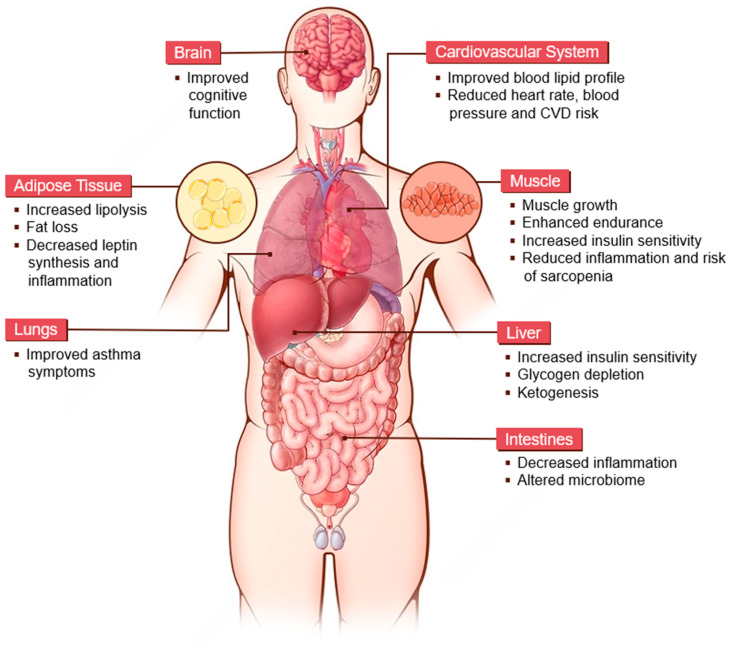
Summary of the key effects of caloric restriction on metabolic homeodynamics in the human body. This figure illustrates this regimen’s impact on the brain, lungs, cardiovascular system, muscles, liver, intestines, and adipose tissue. Abbreviations: CVD, cardiovascular disease. This figure was drawn using images from Servier Medical Art. Servier Medical Art by Servieris licensed under a Creative Commons Attribution 4.0 Unported License (CC BY) (https://smart.servier.com/citation-sharing/).

At the forefront of these research studies stands caloric restriction (CR), a dietary strategy that consists of a reduction in caloric intake while maintaining the essential energy and nutritional requirements to achieve optimal health while avoiding malnutrition [1,2]. The extent of CR implementation can be tailored to individual preferences, ranging from moderate to severe, spanning reported reduction rates from 10% up to 50% below the baseline calorie consumption necessary for the maintenance of the initial body weight [3,4]. The fundamental goal underpinning CR is to achieve and preserve a stable body weight, thus promoting a state of metabolic equilibrium. CR, as a nutritional intervention, has garnered significant scientific attention for its potential impacts on aging, longevity, and overall health [5]. The premise underlying CR is that, by diminishing caloric consumption while upholding essential nutrient intake, it is possible to manipulate fundamental metabolic processes all throughout the human organism (Figure 1). This approach aims to reduce oxidate stress and inflammation, improve general functions such as insulin sensitivity, and modulate several molecular pathways, with the ultimate goal of delaying aging, and subsequently age-related diseases, and extending quality of life [6,7]. Within the different organ systems, CR has been shown to improve physiological functions in various ways. In the central nervous system, CR is able to improve the brain’s cognitive performance by enhancing synaptic plasticity and upregulating brain-derived neurotrophic factor (BDNF), a critical modulator of learning, working memory, and overall neurological efficiency [8]. In the cardiovascular system, CR lowers low-density lipoprotein (LDL) cholesterol and triglycerides while increasing high-density lipoprotein (HDL) cholesterol [9], contributing to a lower risk of cardiovascular diseases (CVDs). Regarding the respiratory system, CR has been demonstrated to improve respiratory function by reducing overall systemic inflammation [10]. Additionally, in the skeletal muscle, CR reduces the risk of sarcopenia and contributes to overall muscle growth, resulting in enhanced endurance [11]. In adipose tissue, CR enhances lipolysis and promotes fat loss, alongside improved leptin sensitivity, contributing to appetite regulation and overall energy balance [12]. CR also promotes changes in the gut microbiome, specifically an increase in the diversity of beneficial bacterial species and a reduction in harmful bacteria [13]. Within the liver, CR leads to glycogen depletion, leading to fat oxidation and ketogenesis [14].

While CR is widely recognized for its systemic metabolic benefits, its impact on reproductive health, particularly male fertility, remains underexplored. Male fertility has historically been a neglected area of research, despite a growing global concern over declining sperm quality and increased infertility rates [15,16]. Presently, contributing factors to male infertility include hormonal and endocrine imbalances, genetic abnormalities, testicular dysfunction, and a variety of systemic pathologies. Nevertheless, approximately 10 to 20% of cases remain undiagnosed—known as idiopathic infertility—wherein conventional diagnostic parameters such as sperm quality parameters (concentration, motility, and morphology) fall within normal ranges, and known pathological causes are absent [15]. These cases point to a potential role for less-explored contributors, including molecular and epigenetic mechanisms, as well as lifestyle and environmental causes, such as pollutants and endocrine-disrupting chemicals. Emerging evidence suggests that dietary patterns exert a significant influence on male reproductive health. Namely, diets characterized by a high intake of saturated fats, refined sugars, and ultra-processed foods (Western diet) have been associated with reduced sperm quality and metabolic disturbances [17]. Within this context, CR may or may not represent a novel strategy to support male reproductive health.

In this manuscript, we reviewed the available literature regarding CR, with a primary focus on its effects on male fertility and quality of life. Due to the lack of research regarding the impact of CR on male fertility, particularly in processes such as spermatogenesis and overall testicular function, we hypothesized that many of the effects of this diet on male reproductive health are due to its ability to modulate sirtuin (SIRT) activity. Hence, we also analyzed the impact of SIRTs and overall acetylation on male fertility through their effects on the hypothalamic–pituitary–gonadal (HPG) axis and the spermatogenetic event. The bibliographical database was compiled from September of 2023 to March 2025, and it was obtained using the PubMed, Scopus, and Google Scholar specialized search engines. Key search terms included, but were not limited to, ‘caloric restriction’, ‘sirtuins’, ‘acetylation’, and ‘male fertility’.

## 2. Caloric Restriction and Sirtuins

Metabolic homeostasis represents the end goal of the intricate cellular machinery operating within the human body. This objective is being constantly challenged by both internal and external stimuli, each capable of impinging upon vital cellular processes, such as growth and proliferation. A wide array of factors, such as hormones and nutrients, act as stimulating/inhibiting inputs, thereby eliciting responses through complex signaling pathways. Consequently, metabolic equilibrium requires meticulous calibration between energy intake, expenditure, and storage, allowing surplus energy to be stored in times of abundance (feeding) and mobilizing said stored energy in times of scarcity (fasting). This metabolic balance is only possible due to the existence of a finely tuned signaling network, involving a variety of pathways associated with feeding, such as the mammalian target of the rapamycin (mTOR) pathway and overall insulin signaling, as well as pathways associated with fasting, notably the AMP-activated protein kinase (AMPK) pathway, and the involvement of SIRTs [18,19,20,21].

### 2.1. Sirtuins and Acetylation

Post-translational modifications are pivotal alterations in proteins involved in cell signaling, profoundly influencing the outcomes of metabolic pathways in response to stimuli. The most common modifications are phosphorylation, acetylation, glycosylation, amidation, hydroxylation, methylation, ubiquitination, and carbonylation, which are cell- and tissue-specific regulators, modulating metabolic responses in different targets [22]. Acetylation is modulated by acetyltransferases, notably histone acetyltransferases (HATs) or lysine acetyltransferases, known for acetylating substrates beyond histones [23]. Conversely, the removal of acetyl groups is performed by histone deacetylases (HDACs). This reaction can either activate or deactivate the substrate, which, according to the target cell or tissue, triggers or inhibits biological processes. Notably, histones emerge as major targets for acetylation/deacetylation, intricately modulating their DNA-binding capacity [24]. In general, acetylation decreases chromatin condensation, resulting in higher transcription levels, while deacetylation elicits the opposite effect [25,26].

In mammals, several HDACs have been described and categorized into four classes, based on similarities between each other and their structural homology with yeast *Saccharomyces cerevisiae* counterparts. Class I includes HDACs 1, 2, 3, and 8, resembling yeast Rpd3, Hos1, and Hos2 deacetylases, while class II comprises HDACs 4, 5, 6, 7, 9, and 10, exhibiting homology with *Saccharomyces cerevisiae* HDA1 and Hos3 deacetylases. Class IV encompasses HDAC 11 and its related enzymes, all utilizing Zn^2+^ as a cofactor for deacetylation reactions [27]. Class III, the most extensively studied of all HDAC classes, includes seven SIRTs (SIRT1-7).

SIRTs constitute a group of nicotinamide adenine dinucleotide (NAD^+^)-dependent deacetylases, serving as metabolic sensors with multifaceted roles. First identified in yeast *Saccharomyces cerevisiae* screenings in the 1970s, these deacetylases were originally given different names depending on the screening objective (cmt—change of mating type; mar—mating-type regulator; sir—silent information regulator), with the scientific community eventually adopting Sir as the primary identification [28,29,30]. The enzymes are involved in several biological processes, including redox signaling, aging, stemness, chromatin remodeling, and inflammatory signaling pathways, among many others [31,32,33]. SIRTs’ involvement in reproductive health has been suggested since the early 2000s, when SIRT1 knockout mice showed an impaired reproductive phenotype [34]. Subsequent studies employing knockout strategies in mice have further illuminated the critical role of these proteins in male reproductive health.

SIRTs represented in HDAC class III are homologous to *Saccharomyces cerevisiae* HDA1 and Hos3 deacetylases, operating as NAD^+^-dependent enzymes. This requirement heavily underscores SIRTs’ role as cellular energy sensors, attuned to variations in energy status and triggering the appropriate responses. In different target molecules, SIRTs are responsible for catalyzing the extraction of acetyl groups, which results in a deacylated protein and the production of nicotinamide (NAM) from NAD+ and O-acetyl-ADP-ribose. Elevated NAM concentrations promote negative feedback on SIRTs through non-competitive inhibition, although competitive inhibition has been described for specific sirtuin isoforms [35]. Interestingly, O-acetyl-ADP-ribose has been associated with signaling functions [36]. Moreover, each member of this class is localized in different subcellular regions, with SIRT1, 2, 6, and 7 primarily in the nucleus and SIRT3, 4, and 5 residing in mitochondria [37,38,39,40,41]. Notably, two of the nuclear SIRTs, SIRT1 and 2, may also be found in the cytoplasm, although the precise subcellular distribution of SIRT1 remains contentious [42,43].

### 2.2. Mechanisms of Sirtuin Regulation

Since their discovery, SIRTs have garnered considerable attention from the scientific community, primarily due to their capacity to extend yeast lifespan [44]. The regulation of sirtuin activity and expression occurs at both transcriptional and translational levels, intricately woven into the cellular response. Given their role as energy sensors, SIRTs exhibit heightened expression and activity during fasting, such as in caloric restriction, and conversely are inhibited during feeding states.

Numerous transcription factors have been identified as key modulators of SIRT1 expression, binding to its promoter region. Among them, cAMP response element-binding protein (CREB), forkhead box O (FOXO) transcription factors, poly(ADP-ribose) polymerase (PARPs), and peroxisome proliferator-activated receptors (PPARs) are well-known modulators of SIRT1 expression [45,46,47,48]. Additionally, the hypermethylated in cancer 1 protein (HIC1) is recognized as a suppressor of SIRT1 expression, since when it binds to the promoter region, it downregulates the activity of SIRT1. In particular, HIC1 inactivation leads to increased SIRT1 expression, resulting in p53 inactivation and establishing a noteworthy association with cancer [49].

At the translational level, SIRT1 messenger RNA (mRNA) is regulated by the protein Hu antigen R (HuR) that is capable of binding to RNA and various microRNAs (miRNAs). HuR exerts a stabilizing effect on SIRT1 mRNA by binding it, thereby prolonging its stability and activity. In contrast, several miRNAs have been recently described to be involved in degrading SIRT1 transcripts and inhibiting translation, delineating a sophisticated post-transcriptional regulatory layer [50,51,52,53]. SIRTs also undergo regulation through the formation of complexes with other proteins, such as deleted in breast cancer 1 (DBC1) and active regulator of SIRT1 (AROS). AROS forms a complex with SIRT1, binding to its N-terminus and augmenting SIRT1 deacetylase activity, albeit contingent on experimental conditions and biological context [54]. Conversely, DBC1 binds to the catalytic domain of SIRT1, impeding deacetylation, particularly under conditions of genotoxic stress [55]. Intriguingly, given SIRTs’ pivotal role in post-translational modifications, their own expression is subject to regulation through such modifications. Phosphorylation and sumoylation stand out as exemplary instances of modifications instrumental in governing SIRT1 expression [56,57,58,59,60].

#### Regulation Through Substrate Availability

**Figure 2:**
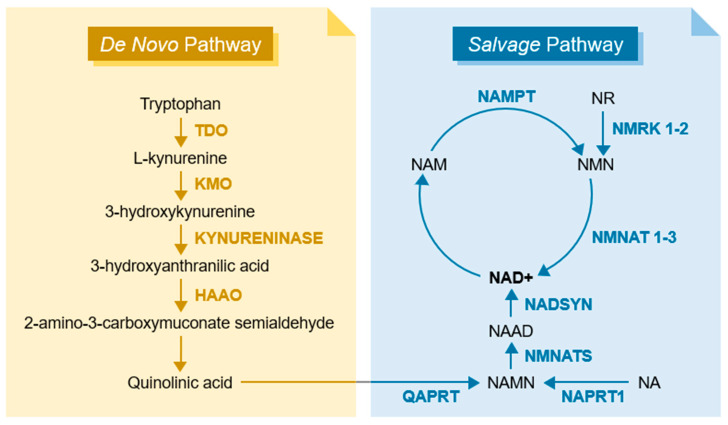
De novo and salvage pathways of NAD^+^ synthesis. In the de novo pathway, also known as the kynurenine pathway, a cascade of enzymes converts tryptophan into quinolinic acid, which is able to be converted to NAD^+^. Conversely, in the salvage pathway, NNAM and NA are recycled using key enzymes such as NAPRT and NAMPT, ultimately leading to the regeneration of NAD^+^. Abbreviations: TDO, Tryptophan 2,3-Dioxygenase; KMO, Kynurenine 3-Monooxygenase; HAAO, 3-Hydroxyanthranilate 3,4-Dioxygenase; QAPRT, Quinolinate Phosphoribosyltransferase; NAMN, Nicotinic Acid Mononucleotide; NAPRT, Nicotinate Phosphoribosyltransferase; NA, Nicotinic Acid; NMNAT, Nicotinamide Mononucleotide Adenylyltransferase; NAAD, Nicotinic Acid Adenine Dinucleotide; NADSYN, Glutamine-Dependent NAD(+) Synthetase; NAD^+^, Nicotinamide Adenine Dinucleotide; NAM, Nicotinamide; NAMPT, Nicotinamide Phosphoribosyltransferase; NMN, Nicotinamide Mononucleotide; NMRK, Nicotinamide Riboside Kinase; NR, Nicotinamide Riboside.

The many functions of SIRTs are heavily influenced by the pools of NAD^+^. NAD^+^ serves as a critical cofactor in various signaling pathways, and its availability is contingent upon the metabolic state of the organism [61]. During feeding states, characterized by elevated glucose levels after a meal, NAD^+^ levels are converted to NADH during glycolysis and the Krebs cycle, and hence are diminished. In contrast, after food ingestion, absorption, and storage, resulting in diminished glucose levels, the oxidation of NADH to NAD^+^ occurs, consequently augmenting sirtuin activity. The synthesis of NAD^+^ can occur through two distinct metabolic pathways: de novo pathways and salvage pathways, the latter of which recycles already existing components for NAD^+^ formation (Figure 2).

De novo synthesis, also known as the kynurenine pathway, uses the essential amino acid tryptophan to produce quinolinic acid [62]. This compound is then converted to nicotinic acid mononucleotide (NAMN), which serves as a substrate for nicotinamide mononucleotide adenylyltransferase (NMNAT) enzymatic reactions, mainly occurring in the liver and kidney, which convert NAMN into nicotinic acid adenine dinucleotide (NAAD). Finally, the enzyme glutamine-dependent NAD^(+)^ synthetase (NADSYN1) converts NAAD into NAD^+^ [63]. Salvage pathways utilize three distinct precursors for NAD^+^ production: nicotinic acid (NA), NAM, and nicotinamide riboside (NR) [64]. These precursors, obtained from dietary vitamin B3 or produced by NAD^+^-consuming enzymes, undergo distinct enzymatic reactions. The enzyme nicotinate phosphoribosyltransferase 1 (NAPRT1) converts NA to NAMN, subsequently following a similar pathway as the de novo synthesis via NMNAT enzyme activity. Afterwards, nicotinamide riboside kinase 1 and 2 (NMRK 1-2) transform NR into nicotinamide mononucleotide (NMN), and further, NMN is converted to NAD^+^ through the involvement of nicotinamide-nucleotide adenylyltransferases 1 to 3 (NMNATs 1-3). Additionally, nicotinamide phosphoribosyltransferase (NAMPT) converts NAM to NMN [65].

The existence of the de novo pathway raises questions regarding its necessity, given that the utilization of NAD^+^ does not change the overall coenzyme levels when in oxidation–reduction reactions. However, NAD^+^ is also engaged in various post-translational modifications, such as deacetylations mediated by SIRTs, leading to NAD^+^ consumption and the production of NAM in deacetylation reactions. Consequently, this results in reduced intracellular NAD^+^ levels. The generated NAM contributes to both salvage and de novo pathways, ensuring the availability of the NAD^+^ cofactor when needed. Furthermore, a noteworthy interplay emerges between NAD^+^-consuming enzymes, exemplified by the synergy between SIRTs and PARPs [66,67,68]. PARPs, crucial for DNA repair and genomic stability, similarly rely on NAD^+^ as a cofactor for the formation of poly(ADP-ribose) chains, functioning as the signal for further enzymes capable of DNA repair [69]. Studies have demonstrated that the deletion of Parp1 and Parp2 activates SIRT1, elevating intracellular NAD^+^ levels and alleviating the repressive effect exerted by PARPs on SIRT1 expression [48,67]. Remarkably, this interaction has been implicated in protecting against diet-induced obesity by enhancing energy expenditure.

### 2.3. Sirtuins as Metabolic Regulators

Whole-body metabolism hinges upon the food one consumes. While an individual’s waking hours are not entirely devoted to eating, the body demands a continual influx of energy, primarily relying on glucose as a metabolic fuel [70]. The nutrients assimilated from the diet, encompassing carbohydrates, fats, and proteins, undergo digestion and subsequently enter the bloodstream. Peripheral tissues, including the liver and adipocytes, promptly take up these nutrients for immediate energy usage or storage. This orchestrated process serves to cater to the body’s energy demands during periods of fasting, exercise, or starvation. In a fed state, elevated glucose levels in the bloodstream prompt β-pancreatic cells to release insulin, which orchestrates glucose uptake in tissues such as white adipose tissue, muscle, and the glucose-dependent brain, fueling energy production through glycolysis. Excessive energy is channeled into glycogen production, efficiently storing glucose in liver and muscle cells. Conversely, during fasting states, glucagon is released from alpha cells in the pancreas as a response to diminished glucose levels. Functioning in direct opposition to insulin, glucagon stimulates hepatic glucose production to meet the energy requirements of other tissues. This process unfolds through sequential events such as glycogenolysis and gluconeogenesis [71].

Numerous studies have elucidated the pivotal role of SIRTs in these metabolic processes, aiming to achieve energy homeostasis [72,73,74]. SIRTs, acting as key regulators, participate in fine-tuning the delicate balance between energy utilization and storage, thereby contributing to the overarching goal of maintaining glucose equilibrium within the body.

#### 2.3.1. Sirtuins and Insulin Signaling

SIRT1 has been prominently linked with insulin secretion and sensitivity [73,75,76]. Numerous studies have highlighted SIRT1’s capacity to modulate critical metabolic pathways in β-pancreatic cells [77]. One of the most significant mechanisms by which SIRT1 exerts its influence is through inhibiting uncoupling protein 2 (UCP2) expression, as shown in a study of pancreatic cells of healthy mice. UPC2, situated within the mitochondrial inner membrane, possesses the ability to dissipate the proton gradient generated during oxidative phosphorylation, which is required for adenosine triphosphate (ATP) synthesis. This implicates SIRT1 in enhancing insulin secretion by transcriptionally repressing UCP2 levels. This inhibition facilitates ATP production through the electron transport chain, thereby augmenting the efficiency of mitochondrial respiration [75,77,78]. The subsequent rise in ATP levels leads to the closure of ATP-sensitive potassium channels, which in turn triggers membrane depolarization and activates calcium channels, allowing calcium influx into the cell. This subsequent increase in calcium is crucial for the exocytosis of vesicles containing insulin, resulting in its secretion into the bloodstream [75,77].

SIRT1 activation also regulates glucose homeostasis in insulin-sensitive organs, acting as a protective shield against insulin resistance [79]. Intriguingly, diabetic patients exhibit SIRT1 inhibition by microRNAs, triggering hepatic insulin resistance [80]. Experiments involving SIRT1 activators and transgenic SIRT1 mice emphasize the correlation between SIRT1 and lower insulin and fasted glucose levels, underscoring its pivotal role in fostering insulin sensitivity [81,82]. Notably, SIRT1 orchestrates the expression of multiple gene transcription factors, thereby contributing to insulin secretion [83].

Remarkably, alterations in SIRT3 expression manifest in the skeletal muscle of mice afflicted with type 1 and type 2 diabetes. This shift in SIRT3 expression exerts regulatory control over mitochondrial ATP and reactive oxygen species (ROS) production, which in turn alters insulin signaling through the same mechanism explained above [84].

Recent investigations have shed light on the significant involvement of SIRT4 in regulating insulin secretion within β-pancreatic cells under the normal dietary conditions of a standard chow diet. In these scenarios, SIRT4 catalyzes the ADP-ribosylation of glutamate dehydrogenase (GDH), leading to the downregulation of its enzymatic activity. GDH, a pivotal player in glutamate and glutamine metabolism, contributes to ATP generation and, subsequently, insulin secretion [85,86]. Mice deficient in SIRT4 or subjected to 40% caloric restriction exhibit elevated GDH activity, underscoring the crucial role of SIRT4 in modulating insulin secretion [85].

This intricate interplay of SIRT1, SIRT3, and SIRT4 in regulating insulin secretion and sensitivity suggests a multifaceted orchestration within pancreatic cells and insulin-sensitive organs. These insights shed light on the intricate regulatory mechanisms that influence glucose homeostasis. As our comprehension of the dynamic role of SIRTs in metabolic processes expands, ongoing research may provide valuable insights into the modulation of these molecular pathways for a deeper understanding of diabetes and related metabolic phenomena.

Additionally, the known regulatory influence of sirtuins on a variety of metabolic processes through insulin signaling extends its effects on male reproductive health. As is well known, insulin is critical for testicular function, with studies showing insulin receptors on both Leydig and Sertoli rodent cells, where it enhances testosterone production and nurtures the development of germ cells, respectively [87,88]. Hence, disruption of insulin signaling, namely through a deficit of SIRT or impairment of its activity, has been linked to impaired testicular function and male fertility [89]. Therefore, the metabolic benefits conferred by SIRT activation, particularly in enhancing insulin signaling and reducing oxidative stress, may extend to preserving male fertility and testicular health.

#### 2.3.2. Sirtuins and Glucose Metabolism

Glucose metabolism is intimately tied to SIRT activity across several checkpoints that range from insulin regulation, as seen above, to glucose homeostasis and regulation of key processes such as gluconeogenesis and glycolysis. In the case of SIRT1, its overexpression in mice pancreatic β-cells has been shown to improve glucose tolerance and response through enhancing the cell’s insulin secretion [77]. Additionally, this overexpression also provides the cell with survival advantages, protecting it from damage incurred during apoptosis, which has proven to worsen prognosis in a variety of cancers [90]. SIRT1 also assumes a pivotal role in modulating glucose production in the liver. One facet of this regulation involves the reduction in glucose production by SIRT1, achieved through the deacetylation of the CREB-regulated transcription co-activator 2 (CRTC2), which subsequently undergoes degradation, resulting in diminished glucose production [91]. Furthermore, SIRT1 promotes glucose production by deacetylating FoxO1 and peroxisome proliferator-activated receptor gamma coactivator-1 alpha (PGC-1α) [73,92]. Notably, the transcriptional activity of FoxO1, which facilitates the expression of genes associated with hepatic glucose production, is promoted with its deacetylation [92]. Simultaneously, the deacetylation of specific lysine residues within PGC-1α activates nuclear genes integral to the gluconeogenic process [73]. Controversy may arise from the seemingly contradictory roles of this enzyme, but it is the pairing between the upregulation and downregulation of the two glucose pathways that leads to an intricate balance between these regulatory mechanisms, which may yield discrete results under different metabolic conditions. In the context of male fertility, the interplay between SIRT1 and PGC-1α, and even SIRT3, is able to regulate processes such as antioxidant defenses, glycolytic activity, and lactate synthesis, which are essential throughout spermatogenesis.

Beyond its capacity in promoting glucose production, SIRT1 assumes a critical function in reducing glucose consumption by inhibiting glycolysis, particularly under fasting conditions, such as during CR. This inhibitory effect is achieved through a lower expression of glycolytic genes modulated by PGC-1α and hypoxia-inducible factor 1α (HIF-1α), which promote oxidative metabolism [73]. Additionally, SIRT1 deacetylates phosphoglycerate mutase-1 (PGAM-1), a key enzyme involved in glycolytic processes. This process, in turn, leads to the attenuation of the catalytic activity of PGAM-1, downregulating various steps within glycolysis, impacting the cellular metabolism and related functions [93]. Within gluconeogenesis, SIRT1 plays a role in improving insulin sensitivity during fasting through its interactions with key regulators such as glucose transporter type 4 (GLUT4), nuclear factor kb (NFkB), and tyrosine phosphatase 1B gene (PTP1B), which negatively regulates the insulin signaling pathway [94].

As for SIRT2, in the context of gluconeogenesis, this SIRT modulates and stabilizes phosphoenolpyruvate carboxykinase (PEPCK-C), a key enzyme in numerous metabolic processes, which catalyzes certain irreversible reactions crucial to gluconeogenesis [95]. Notably, during caloric restriction, the increased expression of SIRT2 in mice has a role in regulating adipose tissue function [92]. Intriguingly, adipose tissue exhibits elevated levels of both SIRT2 protein and mRNA, with their overexpression playing a significant role in adipocyte differentiation. SIRT2, in its regulatory capacity, can inhibit adipogenesis through its interaction with FoxO1. This inhibition is achieved by reducing the acetylation of FoxO1, thereby enhancing its affinity to bind to peroxisome proliferator-activated receptor gamma (PPARγ) or its promoter, which results in the inhibition of PPARγ activity or a reduction in its transcription [92,96]. Through these intricate molecular mechanisms, SIRT2 can influence both lipid and glucose metabolism. On the other hand, SIRT4 has a contrasting effect on healthy pancreatic β-cells to that of SIRT1. When mitochondrial SIRT4 is selectively expressed within these cells, it induces the ADP-ribosylation of GDH, working as a negative regulator of the cell’s insulin secretion [97,98].

Diverging from its SIRT counterparts, SIRT5 is most renowned not for its deacetylase activity, but rather for its proficiency in glutarylation, demalonylation, and desuccinylation processes [99,100]. SIRT5 is able to regulate intermediary metabolic processes, namely glycolysis, as evidenced by the notable reduction in glycolytic rate in primary hepatocytes lacking SIRT5. Additionally, SIRT5 also manages to regulate the catalytic activities of glyceraldehyde-3-phosphate dehydrogenase (GAPDH) and 3-hydroxy-3-methylglutaryl-CoA synthase 2 (HMGCS2), critical enzymes within various glycolytic processes and ketone body production, respectively. This control is achieved through the demalonylation and desuccinylation of specific lysine residues within the protein sequences of both enzymes [101,102].

SIRT6 exhibits a multifaceted role in modulating insulin signaling and glucose homeostasis. Firstly, SIRT6 serves as an inhibitor of the insulin receptor (IR) and insulin receptor substrates 1 (IRS1) and 2 (IRS2), acting as a negative regulator of AKT phosphorylation, which results in the suppression of AKT and insulin signaling. Additionally, SIRT6 is capable of inhibiting GLUT1 and GLUT4. The combined effect of SIRT6 on IR, IRS, AKT, and glucose transponders empowers this SIRT to be able to suppress pathways associated with insulin signaling, thereby contributing to the maintenance of glucose homeostasis [103]. In a particular study, it was shown that when SIRT6 is deficient within the pancreatic cells of healthy mice, there is a subsequent upregulation of GLUT1 and GLUT4 expression. The abundance of these glucose transporters, in turn, increases glucose uptake, resulting ultimately in a state of hypoglycemia [104]. This deficit also increases the acetylation of histone H3 lysine 9 (H3K9), accompanied by heightened transcriptional and promoter-binding activity of c-Jun, which leads to the activation of multiple insulin-like growth factor (IGF) signaling pathways [105]. Moreover, SIRT6 is also capable of downregulating the expression of a variety of glycolytic genes, resulting in a reduction in glycolytic rate, as well as certain transcription factors, notably hypoxia-inducible factor 1α (HIF-1α), which responds to nutrient stress [105,106]. In mice adipocytes and muscle cells deficient in SIRT6, HIF-1α levels are elevated, alongside diminished mitochondrial respiration and augmented glucose uptake and an overall increase in glycolysis [105]. SIRT6 also exerts control over glyconeogenesis by enhancing the activity of General Control Non-repressed Protein 5 (GCN5). This activation leads to the catalysis of PGC-1α and PPARγ. Consequently, these factors downregulate several glyconeogenic enzymes, including glucose-6-phosphatase (G6P) and phosphoenolpyruvate carboxykinase (PEPCK-C), thereby inhibiting gluconeogenesis and repressing hepatic glucose production [107].

#### 2.3.3. Sirtuins and Lipid Metabolism

SIRTs also play a crucial role in various aspects of lipid metabolism, spanning from biosynthesis to storage and utilization. Notably, one of the most important pathways for these processes is insulin secretion. Insulin increases the number of fatty acids that are stored within white adipose tissue while safeguarding them from oxidative processes in the skeletal muscle or liver. Moreover, the impact of SIRTs transcends the regulation of basal metabolic processes and affects the response from stress-inducing stimuli. During stress-inducing events, such as CR, fasting, and many others, SIRTs are prompted to exert a variety of adaptive responses [108].

SIRT1 is able to regulate various genes (and their resulting factors) that partake in lipid metabolism. Specifically, under fasting conditions, whether short-term or prolonged, the body shifts its focus from pathways associated with lipid synthesis and storage to lipolysis [109]. SIRT1 contributes to this metabolic transition by acting as a negative regulator for Sterol Regulatory Element-Binding Proteins 1 (SREBP-1) and 2 (SREBP-2) during fasting. SREBPs, sequestered at the nuclear envelope, are activated when lipid levels, particularly sterols, are diminished. SIRT1 prevents the activation of SREBPs; without this regulatory mechanism, activated SREBPs are cleaved from the nuclear envelope and translocate to the nucleus, augmenting the transcription of enzymes that contribute to sterol synthesis [110]. Additionally, SIRT1 is further able to inhibit fatty acid synthesis when activated by compounds such as resveratrol. This activation leads to an increase in AMPK activity, a cellular energy sensor that influences cellular metabolism and energy balance [92]. By suppressing processes associated with lipid synthesis and promoting their breakdown for energy production during periods of fasting, SIRT1 integrates seamlessly within the broader metabolic machinery.

In white fat tissue, SIRT1 orchestrates a complex regulatory network by repressing mediators for retinoic acid and thyroid hormone receptor (SMRT) and interacting with PPARγ and its nuclear receptor co-repressors (NCoR). This culminates in the formation of the SIRT1/PPARγ/NCoR complex, suppressing PPARγ activity by interacting with the promoter regions of different PPARγ target genes, inhibiting their transcription and subsequently several lipolysis and fatty acid accumulation processes. In this way, SIRT1 is able to meddle with energy production processes within the cell to favor mobilizing energy from white and brown adipose tissues [111]. During fasting conditions, SIRT1’s deacetylation of PGC-1α activates genes pivotal for the oxidation and usage of fatty acids [110].

Beyond its role in metabolic homeostasis, SIRT1 is also beneficial to longevity across various species [112]. Mice genetically modified to have a knockout of the SIRT1 gene exhibit lower levels of HDL, triglycerides, and total plasma cholesterol. Additionally, SIRT1 acts as a positive regulator for liver X receptor (LXR), deacetylating LXR on specific lysine residues, promoting its ubiquitination and subsequent degradation. LXR is a key player within cholesterol metabolism, and its degradation promotes cholesterol efflux from the cells. Furthermore, the activation of LXR mediated by SIRT1 elicits numerous beneficial effects, such as the inhibition of cholesterol uptake in the intestine and anti-inflammatory responses [113].

SIRT4 also has an important role in modulating lipid metabolism through the increased expression of associated enzymes [114]. Specifically, in mice models featuring genetic modifications resulting in the absence of the SIRT4 gene, a notable upregulation is observed in the expression of peroxisome proliferator-activated receptor alpha (PPARα) target genes. These genes are intricately associated with the process of fatty acid catabolism [115].

SIRT6 has been proven to regulate a variety of processes integral to lipid homeostasis within the cell. Studies have shown that mice engineered to overexpress SIRT6 exhibit improvements in glucose tolerance, insulin secretion, and overall blood lipid profiles when subjected to a high-fat diet, as well as a reduction in visceral fat accumulation [116]. One of the important associated mechanisms is the inhibition of SREBP, which plays a central role in cholesterol homeostasis. SIRT6 achieves this regulatory control by deacetylating histone H3, a process that inhibits SREBP expression. In SIRT6-deficient mice, FoxO3 tasks SIRT6 to deacetylate histone H3 at specific lysine residues within the promoter region of the Srebp2 gene, thereby suppressing the expression of SREBP-2 and its downstream target genes [117]. Moreover, SIRT6 exerts an additional layer of regulation by deacetylating H3K9 on the promoters of various genes associated with lipid metabolism. In the case of a SIRT6 deficit, the altered expression of these genes leads to the development of a fatty liver phenotype in the above-mentioned mutant mice expression of genes associated with β-oxidation [118].

While SIRT7 appears to possess a pronounced role in regulating lipidic metabolism, particularly in the liver, a consensus on its regulatory mechanisms has not yet been reached. Two studies involving SIRT7 knockout mice showed increased triglyceride levels, heightened expression of lipogenic genes, and the development of liver steatosis. Remarkably, these studies propose distinct mechanisms underlying these metabolic alterations [119,120]. In one of the studies, it was suggested that c-Myc, a regulator of cell proliferation and metabolism, recruits SIRT7 to suppress stress in the endoplasmic reticulum (ER). In hepatocytes lacking SIRT7, unresolved ER stress is followed by liver steatosis, which later evolves to dyslipidemia [119]. Conversely, the second study proposes that SIRT7 deacetylates a well-known regulator of mitochondrial genes, GA-binding protein 1 (GABP1), resulting in alterations in mitochondrial homeostasis [120]. However, a third study challenges this paradigm by demonstrating that SIRT7 knockout mice exhibit resistance to obesity, improved glucose tolerance, and reduced liver steatosis, accompanied by diminished triglyceride levels. In this study, it was proposed that SIRT7 interacts with a DDB1-CUL4-associated factor 1 (DCAF1)/damage-specific DNA binding protein 1 (DDB1)/cullin 4B (CUL4B) E3 ubiquitin ligase complex. This interaction inhibits the degradation of testicular receptor 4 (TR4), thereby mitigating the uptake of fatty acids and the synthesis and subsequent storage of triglycerides [121]. Additionally, SIRT7 has been implicated in the regulation of hypoxia-inducible factor-1 alpha (HIF-1α) and HIF-2α, downregulating their transcriptional activity. This modulation decreases protein levels, namely those associated with energy metabolism [122].

## 3. Impact of Caloric Intake on Male Reproductive Health

The current lifestyle of Western societies is characterized by high-fat diets and sedentary behaviors, leading to the accumulation of energy within the body. These unnatural behaviors disrupt metabolic homeostasis, exerting pressure on biological systems and predisposing them to the development of various disorders, notably metabolic syndrome and diabetes mellitus. Simultaneously, there has been a noticeable decline in male fertility parameters, particularly sperm parameters, which have reached historic lows within the last few decades [16]. Several studies have shown that male fertility is susceptible to metabolic shifts caused by toxins and a variety of disorders [123,124,125,126,127]. Metabolism is pivotal to spermatogenesis as it directly influences the formation of spermatozoa, thereby intricately linking metabolic health to the fertility potential of an individual. Somatic Sertoli cells are a central piece of spermatogenesis as they are solely responsible for supporting germ cells physically and nutritionally [128]. A comprehensive body of research has shown that in metabolic disorders, testicular metabolism is expectedly dysregulated, resulting in compromised sperm parameters [129,130,131]. This network of metabolic checkpoints governing the balance between energy production through the consumption of lipids, proteins, and carbohydrates, and energy storage is, as outlined above, tightly coordinated and managed by metabolic sensors, in which SIRTs play an important role.

### 3.1. Male Fertility Under the Influence of Caloric Restriction

Some studies have looked into the effect that CR has on male fertility, encompassing assessments of the HPG axis, sperm quality, and various testicular parameters. However, a consensus remains elusive regarding the affirmative influence of CR on male fertility, especially when considering interspecies variations. In a study employing Wistar rats subjected to a standardized CR regimen for 28 days, comprising a 30% caloric reduction compared to a control group fed ad libitum, nuanced effects were observed in the hormonal profile of the animals and in the metabolic profile of the testes, alongside sperm quality outcomes [132]. On one hand, no discernible alterations were noted in glycolytic metabolism; rather, a preference for using lipids as an energy source emerged, specifically triglycerides and phospholipids, aligning with anticipated CR metabolic shifts. Simultaneously, diminished levels of oxidative stress markers were observed, corroborating established CR effects. With relation to SIRTs, only the expression of SIRT1 mRNA was assessed, and it showed a slight yet not statistically significant increase. On the other hand, rats exposed to CR exhibited expected reductions in plasma leptin, coupled with elevated ghrelin and glucagon-like peptide 1 (GLP-1) levels, indicative of a state of diminished weight and potentially heightened hunger, concomitant with the characteristic metabolic adaptations of reduced food intake. Additionally, amino acid availability linked to protein synthesis suffered a reduction, underscoring systemic alterations in response to the dietary regimen. Notably, organ systems displayed a shift from biosynthesis to energy conservation processes, consistent with CR expectations. More importantly, the study evidenced an increase in sperm head defects, which severely impact sperm quality [132]. The most noted increase was in decapitated spermatozoa, accompanied by a slight, non-significant decrease in sperm concentration and viability. These changes were strongly correlated with a reduced fertility potential, which translates in practice to a correlation with lower pregnancy rates. This contrasts with the otherwise positive aspects of CR on the human body, where the controlled caloric consumption resulted in tighter metabolic control. However, in fertility-associated processes, such as spermatogenesis, there is an undeniable high energy cost, which CR, by reducing caloric intake, is unable to reach, sacrificing fertility potential and overall testicular function.

Contrastingly, a study into aged Wistar rats yielded more promising outcomes. Apart from CR’s well-established role in weight maintenance and life expectancy extension in older models, noteworthy enhancements were observed in parameters associated with glucose homeostasis and insulin secretion, contrary to what was previously reported [133]. Notably, CR attenuated the age-related deterioration of pancreatic cells, sustaining glucose homeostasis. Importantly, testosterone concentrations mirrored those of significantly younger rats, suggesting a potential role for CR in prolonging the reproductive period, as has long been hypothesized. CR further induced improvements in male sexual behavior, manifested as a reduction in 17β-estradiol levels in two groups of rats subjected to 15% and 35% of CR, compared to the control group, positively impacting mood, sexual function, and sperm production positively [133].

Exploration of the effects of CR on male fertility extends to studies involving *Rhesus macaques*, revealing promising insights that not only show potential benefits but also affirm the lack of adverse impacts on reproductive parameters [134,135]. A comprehensive study conducted at the University of Maryland implemented a 30% CR regimen on *Rhesus macaques*, commencing at 4 years of age, in comparison to a control group fed ad libitum. Seven years into the dietary intervention, the influence of CR on gene expression is pertinent to testicular functions and within the HPG axis. Noteworthy findings emerged with the downregulation of the glycoprotein hormone alpha subunit (CGA) gene, which encodes the alpha subunit of various glycoprotein hormones, and TSH receptor mRNA in the pituitary gland. However, the absence of collateral changes in the broader hormonal profile mitigates the likelihood of these variations originating from perturbations of the HPG axis, pointing instead to potential alterations in other metabolic functions [135].

Concurrent assessments of testicular function and spermiogram parameters in the same subjects mirrored the gene expression results, with the sole notable difference being an increase in the minimum daily testosterone levels in CR macaques compared to the control group. This isolated change, unaccompanied by alterations in other HPG axis parameters, suggests that the observed testosterone increase may be attributed to distinct physiological shifts in alternative systems, such as the liver. The augmented hepatic activity could potentially enhance the conversion of dehydroepiandrosterone into testosterone. Furthermore, the state of CR might facilitate the redirection of energy toward critical physiological functions, consistent with prior observations regarding sirtuin expression. This could explain the overall diminished variation in the majority of sperm parameters, indicative of a more tightly regulated homeostasis in the context of CR [134].

Regardless of the type of dietary restriction an individual may choose to follow, we cannot rule out the possibility of environmental contaminants (such as heavy metals, microplastics, or pesticides) being present in the diet. Their effects (often negative) on male fertility have been documented; however, information specifically regarding the modulation of SIRTs is very limited, and to the best of our knowledge no human studies are available. Cadmium (Cd), a heavy metal and a well-known environmental contaminant, is known to affect male fertility. In mice models, Cd downregulates SIRT1 mRNA expression in Leydig cells [136], and decreases the protein levels of SIRT1 in testicular tissue [137]. Cd was also shown to suppress the protein expression of SIRT3 in rat testis [138]. A cell line from spermatids (GC-2) presented lower protein levels of SIRT1 after exposure to arsenic [139]. Pesticides are an almost inevitable contaminant in the diet; iprodione is widely used for fungal control and, in the testicular tissue of male Sprague Dawley rats exposed to iprodione, mRNA expression of SIRT1 was decreased when compared to non-exposed rats [140]. The presence of microplastics in organisms has led to rising concerns about their negative effects. In a study with C57BL/6 male mice and mouse spermatogonia-derived GC-1 cells, SIRT1 protein levels were downregulated after polystyrene nanoplastic exposure in vitro and in vivo [141]. A study using Sirt1 knockout mice exposed to fine particulate matter (PM_2.5_) highlights that PM_2.5_ induces ferroptosis through the SIRT1/HIF-1α signaling pathway, thereby inhibiting testosterone synthesis in males [142]. Di(2-ethylhexyl) phthalate (DEHP) is a microplastic and widespread environmental contaminant; in a study with SPF (Specific Pathogen-Free) male ICR mice, mRNA levels of Sirt1 were reduced in the DEHP exposure group [143].

In the context of SIRTs, there is an absence of studies to elucidate the impact of CR on these enzymes and their subsequent influence on the male reproductive system and fertility. Moreover, in the context of SIRTs, a paucity of studies exists elucidating the impact of CR on these enzymes and their subsequent influence on the male reproductive system and fertility. Nevertheless, it is plausible that CR modulates male fertility through the activity of various SIRTs, particularly SIRT1 [144,145]. Compounds known to activate SIRT1, such as resveratrol, have demonstrated notable health benefits in murine models [144,145,146]. Resveratrol, a naturally occurring polyphenol found in plants like berries and nuts and products such as red wine, exhibits anti-inflammatory and antioxidant properties. Beyond its reproductive implications, resveratrol is associated with multifaceted health benefits, encompassing a reduction in blood pressure, neuroprotection, and potential anticancer effects [147,148]. As a supplement, resveratrol has been shown to activate genes, including SIRT1, implicated in extending lifespan. However, the precise mechanisms underlying this activation remain a subject of ongoing investigation. One proposed pathway suggests that resveratrol may indirectly activate SIRT1 by binding to and inhibiting phosphodiesterases, preventing the breakdown of cyclic adenosine monophosphate (cAMP). Elevated cAMP levels, in turn, activate AMPK, ultimately stimulating SIRT1 [144]. Alternatively, the activation of SIRT1 by resveratrol has been shown, in a direct manner, in studies employing fluorophore-labeled peptides, establishing that resveratrol SIRTs have the capacity to directly activate both SIRT1 and SIRT5. Consequently, activated SIRTs, including SIRT1, may exert a range of effects on male fertility and testicular function, whether through direct or indirect mechanisms [145].

### 3.2. Sirtuins and the Hypothalamic–Pituitary–Gonadal Axis

As previously mentioned, SIRTs wield considerable influence over diverse cellular processes, strongly influencing the male reproductive system. Of particular significance is SIRT1, which scores considerable influence in regulating spermatogenesis and overall male fertility through modulation of the HPG axis. Under physiological conditions, the gonadotropins luteinizing hormone (LH) and follicle-stimulating hormone (FSH) assume key roles as modulators of spermatogenesis. FSH, binding to its cognate receptor on Sertoli cells, regulates the continued development and functionality of these cells [129]. Simultaneously, LH engages its corresponding receptor on Leydig cells, promoting the biosynthesis of testosterone, which, in turn, regulates spermatogenesis by interacting with its androgen receptor on Sertoli cells [129,149].

**Figure 3:**
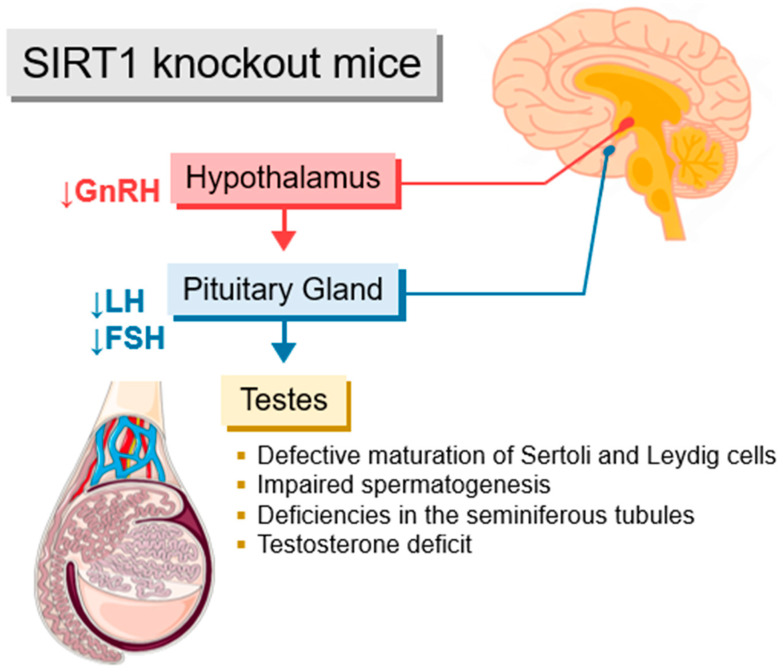
Effects of SIRT1 deficit in SIRT1 knockout mice on the HPG axis and testicular function. In mice with a knockout of the SIRT1 gene, the resulting reduction in GnRH secretion leads to lower LH and FSH levels in the pituitary gland. Subsequently, the reduced levels of gonadotropic hormones impair gonadal function by generating a deficit in testosterone, defects in Sertoli and Leydig cells, and within the development of spermatozoa. Abbreviations: FSH, follicle-stimulating hormone; GnRH, gonadotropin-releasing hormone; LH, luteinizing hormone. This figure was drawn using images from Servier Medical Art. Servier Medical Art by Servieris licensed under a Creative Commons Attribution 4.0 Unported License (CC BY) (https://smart.servier.com/citation-sharing/).

SIRT1 was shown to be abundantly expressed within the hypothalamus [128,150]. In SIRT1 knockout mice, there is a discernible reduction in gonadotropin-releasing hormone (GnRH) secretion, culminating in diminished LH and FSH levels (Figure 3). The reported disruption manifests itself in abnormalities within the maturation of Leydig and Sertoli cells, ultimately leading to apoptosis in male germ cells. Furthermore, the perturbation in apoptosis cascades into a deficit in testosterone levels within the testes. Notably, the absence of SIRT1-mediated deacetylation in SIRT-/- mice downregulates the expression of specific genes in Leydig cells that are integral to steroidogenesis, thereby impacting the conversion of cholesterol into steroid hormones, including androgens [130]. Mice genetically engineered with deactivated gonadotropin-encoding genes yield a phenotype similar to the one observed in SIRT-/- mice, emphasizing the interplay between SIRT1 and the HPG axis in governing testosterone biosynthesis [151,152,153,154]. Moreover, SIRT-/- mice demonstrate a failure in the migration of GnRH to the hypothalamus from the vomeronasal organ during embryonic stages, resulting in hypogonadotropic hypogonadism. This condition denotes a diminished function of the gonads, leading to the reduced production of sex hormones and overall impairment of the reproductive function [131].

### 3.3. The Influence of Sirtuins in the Spermatogenetic Event

In mammalian testicular tissue, SIRTs represent a group of enzymes that are abundantly present, with a notable emphasis on SIRT1 [155]. This SIRT is discernibly present in germ cells throughout various stages of spermatogenesis, including spermatids, spermatocytes, and spermatogonia [34,155]. The absence of SIRT1, as observed in SIRT-/- mice, results in a significant reduction and sometimes absence of spermatids, ending spermatogenesis during late prophase I. This interruption leads to a number of structural abnormalities, DNA damage, and other apoptotic features [34,130,156,157,158]. The profound impact of this deficit underscores the direct involvement of SIRT1 in the maturation of germ cells in spermatogenesis [34]. The first possible explanation of this effect is correlated with the regulatory protein p53, and its effect on apoptosis. In cases of SIRT1 deficit, diminished overall cellular deacetylation leads to the upregulation of p53, activated by acetylation [34]. This heightened p53 activity subsequently increases the rate of testicular apoptosis [159,160].

**Figure 4:**
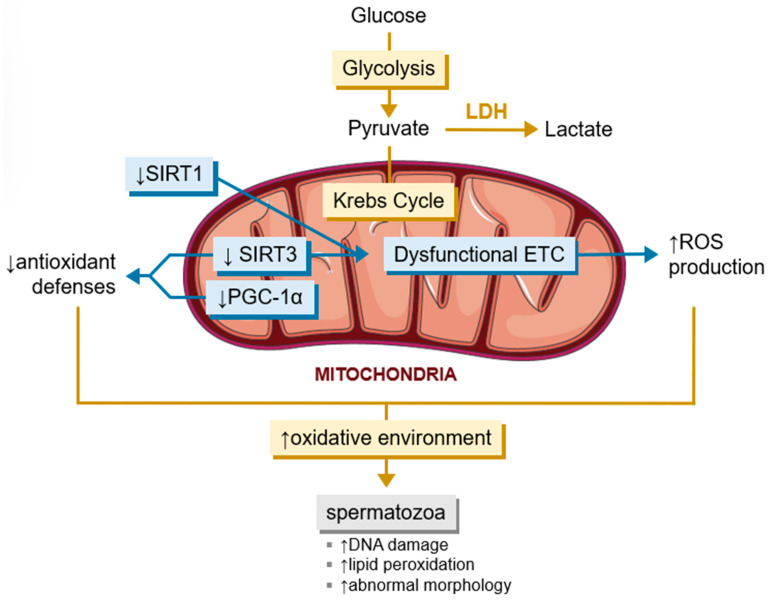
Effects of SIRT1 and SIRT3 deficit on testicular metabolic pathways. SIRT1 and SIRT3 are strong contributors to the cell’s antioxidant defenses. When the levels of these SIRTs are reduced in the testicular environment, the ETC is debilitated within the mitochondria, leading to ROS overproduction. Coupled with lower levels of PGC1α, this deficit ultimately leads to diminished antioxidant defenses, resulting in an increase in oxidative stress within the cells, which affects the spermatozoa by leading to abnormal morphology and DNA damage. Abbreviations: LDH, Lactic Acid Dehydrogenase; PGC-1α, peroxisome proliferator-activated receptor gamma coactivator 1-alpha; SIRT, sirtuin. This figure was drawn using images from Servier Medical Art. Servier Medical Art by Servieris licensed under a Creative Commons Attribution 4.0 Unported License (CC BY) (https://smart.servier.com/citation-sharing/).

SIRT1 is a contributor to the mechanism of cellular defense against antioxidants, alongside SIRT3 and PGC-1α (Figure 4). SIRT1 deacetylates PGC1α, resulting in its activation, subsequently promoting the transcription of the gene that codes for SIRT3, otherwise expressed in mammalian testicular tissue [155,161]. Any disturbances to this pathway have been shown to create problems in the antioxidant defenses and ROS homeostasis in testes [162]. Within the testicular cellular environment, the germ cells membranes possess a high polyunsaturated fatty acid (PUFA) content as a marking characteristic. PUFAs are important for membrane structure because they provide fluidity and are very impactful for the membrane’s ability to fuse with other membranes, which are two characteristics essential for interactions between sperm cells and the ovule, such as acrosomal exocytosis. However, PUFAs make the cells more prone to damage through oxidative stress, namely lipid peroxidation [163,164,165,166]. This oxidative stress emerges as a plausible explanation for the changes occurring in spermatogenesis resulting from SIRT1 deficit. Additionally, a deficit in SIRT3 leads to higher glycolytic activity in the testes, which may hinder testicular metabolism since it leads to the exacerbated production of ROS within the mitochondria [162,167]. This correlation is also seen in cases where the deficit of SIRT1 and SIRT3 has been shown to cause disturbances across the electron transport chain and the mitochondrial pathway of apoptosis, besides the previously mentioned changes in antioxidant defenses [168,169].

Oxidative stress, and the subsequent apoptosis, are possible explanations for DNA damage present in germ cells of SIRT1-/- mice [130,157,158]. These mice also possess defects in various processes associated with the differentiation of germ cells during spermatogenesis, such as histone-to-protamine transition, as well as altered chromatin condensation, increasing their propensity for DNA damage [158]. However, SIRT1 is not the only SIRT involved in the chromatin condensation of sperm, since SIRT6 also strongly influences the activity of protamines. Evidence of this behavior occurs in obese mice subjected to a high-fat diet, which showed decreased SIRT6 expression in spermatids and a resulting poor sperm protamination [170]. Additionally, when a global gene expression analysis was performed in mice deficient in SIRT1, it was revealed that this deficit leads to changes in the expression of many genes associated with the spermatogenic process, as well as in sumoylation, which has been established to be regulated by SIRT1. Besides its widely known effects on transcriptional regulation, stress, protein stability, apoptosis, and overall progression through the cell cycle, sumoylation also plays key roles within spermatogenesis and overall testicular function, namely in spermatogonia, meiotic sex hormone inactivation, and changes within the spermatid nucleus [171,172,173,174,175].

SIRT1 also exerts its influence over structural aspects of the testicular tissue, as well as in spermatogenesis. In SIRT1 knockout mice (SIRT1-/-), besides the deficit in the spermatozoa count, there are also changes to the tubular lumen and seminiferous tubules, which are smaller and structurally abnormal when compared to those of normal mice. Additionally, the expression of reproductive homeobox 5 (RHOX5) is reduced, which is a gene involved in the differentiation process and function of the Sertoli cells.

These changes suggest that a lack of SIRT1 affects Sertoli cell maturation, highlighting SIRT1’s role in their differentiation. Beyond Sertoli cells, SIRT1 also affects the overall development of Leydig cells. SIRT1-/- mice possess testes that present a deficit in adult Leydig cells, as well as in intratesticular testosterone levels, indicating that SIRT1 impacts later stages of Leydig cell maturation and function [130,157]. Additionally, lower levels of male hormones such as FSH and LH have been identified in these mutant mice, which indicates that SIRT1 has at least some level of hypothalamic–pituitary control of the gonads, as mentioned above [130]. SIRT1’s effect on spermatogenesis is shown by the presence of uncommon spermatocytes within the seminiferous tubules in mice deficient in SIRT1, with abnormalities in the number of nuclei and size. This happens due to the absence of SIRT1 acting as a downregulator for genes associated with later stages of meiosis in spermatozoa, which results in the stoppage of spermatogenesis in late prophase. SIRT1 further contributes to genomic integrity by protecting DNA against damage and the resulting apoptosis, which is seen to be widely present in the seminiferous tubules of SIRT1-/- mice [176].

Furthermore, SIRT1 is also strongly impactful in a variety of diseases associated with the male reproductive system. Varicocele is a disease that consists of the enlargement of the veins within the scrotum. Patients with varicocele present an increase in oxidative stress, exhibiting higher than normal levels of ROS, which lead to infertility, as well as a reported SIRT1 deficit in some cases [177]. SIRT1 is notable for its capability to promote antioxidant defenses, so its absence exacerbates the oxidative damage caused by varicocele, which then amplifies the resulting damage to DNA and ultimately leads to infertility [177,178]. Additionally, varicocele can worsen semen parameters, leading to low sperm count (oligozoospermia), bad sperm motility (asthenozoospermia), and overall abnormal sperm morphology (teratozoospermia). Altogether, these conditions constitute what is called oligoasthenoteratozoospermia (OAT). Patients with OAT, particularly when resulting from varicocele, also present a lower expression of SIRT1 in the seminal fluid than fertile men [177]. However, after corrective surgery, the same patients demonstrated a positive increase in SIRT1 levels, as well as improved sperm parameters [179].

Another rarer cause of male infertility is globozoospermia, which involves the presence of an abnormally shaped acrosome in male germ cells, or overall absence, which leads to the presence of spermatozoa with a rounded head, instead of the normal oval shape, making them unable to partake in the reproductive process. SIRT1 is implicated in the regulation of acrosome formation, since its normal deacetylase function is able to deacetylate LC3, a key player in modulating autophagy, allowing it to be redistributed through the cytoplasm in order to partake in the formation of the acrosome. Consequently, a deficit in SIRT1 and its deacetylase activity leads LC3 to be continuously acetylated, and slowly accumulated in the nucleus, unable to move through the cytoplasm to partake in autophagy, which may result in globozoospermia [180].

SIRT3, SIRT4, and SIRT5 constitute a subset of SIRTs exclusively localized within the mitochondria, acting as key regulators in ATP production and diverse metabolic processes [181]. As previously mentioned, SIRT3 is distinguished for its antioxidant properties, alongside SIRT1, as well as its involvement in regulating the production of ATP. This occurs through the targeting proteins within the ATP synthase complex for acetylation. In instances of SIRT3 deficiency, the resultant decline in deacetylase activity impairs the functionality of this complex, culminating in a reduction in ATP synthesis. These alterations are sure to affect many metabolic processes across various cell types, including male germ cells and spermatogenesis. Moreover, additional repercussions of SIRT3 on fertility are underscored by its implications in the aging process [182]. Furthermore, patients afflicted with asthenoteratozoospermia, characterized by reduced sperm motility and abnormal sperm morphology, exhibit diminished levels of both SIRT3 and SIRT1 in their seminal plasma compared to normospermic individuals. Similar to SIRT3, SIRT4’s impact on spermatogenesis and testicular tissue is attributed to its regulatory influence on ATP production, thereby modulating energy and oxidative homeostasis. While the significance of both SIRT3 and SIRT4 is acknowledged, a comprehensive understanding of their specific roles and mechanisms within the testes remains elusive, necessitating further in-depth investigation.

SIRT5 is equally active in the mitochondria, which has the potential to be quite impactful within male fertility, since there are many mitochondrial functions essential to a variety of processes within all cells, particularly germ cells, namely proliferation and apoptosis [183]. Mitochondria, particularly vital in spermatozoa, contribute significantly to sperm motility, notably within the microtubules of the sperm tails [184]. Notably, a deficit in SIRT5 has been identified in the semen of infertile men in comparison to their fertile counterparts, as well as an increase in mitochondrial fragmentation. This perturbation bears the potential to induce dysfunctional mitochondria [181]. Ultimately, research seems to show that all mitochondrial SIRTs are very impactful on the quality of sperm and for the handling of stress within the male reproductive system, strongly impacting fertility [185].

SIRT2 exhibits the ability to deacetylate a lysine residue on α-tubulin within sperm microtubules. This deacetylation process imparts a diverse array of functional properties to microtubules, thereby facilitating their interactions with various proteins and modulating several essential aspects of cellular function [186,187]. Previous investigations have demonstrated that enzymes with deacetylating capabilities targeting α-tubulin, such as HDAC6, contribute to sperm motility. In instances where HDAC6 is deficient, there is an elevation in α-tubulin acetylation, ultimately influencing sperm motility. Consequently, it is plausible to hypothesize that SIRT2 may have a comparable effect on spermatozoa, thereby implicating its role in fertility [27,186].

In SIRT6, a study involving mice subjected to a high-fat diet revealed a diminished expression of SIRT6 within spermatozoa at various stages of spermatogenesis. This reduction in SIRT6 levels resulted in a compromised deacetylating activity, leading to an accumulation of acetylated H3K9. Furthermore, an increased presence of damaged DNA suggests a role of SIRT6 in mitigating DNA damage, either through prevention or by stimulating repair mechanisms, as evidenced in prior studies [170,188]. More recently, SIRT6 has been identified as a critical regulator of spermatogenesis. Through a study on knockout mice, it was demonstrated that spermatogenesis was arrested at the elongated spermatid stage, leading to a significant reduction in mature sperm cells and the presence of malformed acrosomes. Despite exhibiting normal mating behavior, the knockout mice were sterile due to the halted progression of spermatogenesis and increased germ cell apoptosis [189].

In the case of SIRT7, this enzyme is associated with the regulation of transcriptional activity. A deficit in SIRT7 activity, either through knockout models or inhibition of its deacetylase activity, leads to a decrease in the expression of RNA polymerase I and its associated transcriptional processes. Conversely, the opposite effect happens when SIRT7 is overexpressed [37,190]. Analogous to SIRT1, SIRT7 contributes to genome integrity and successful embryonic viability [190]. Although a comprehensive understanding of the roles of SIRT2, 6, and 7 in male fertility and testicular function is currently lacking, these enzymes have great potential to be very impactful within male fertility and overall testicular function, given their considerable impact throughout cellular metabolism.

## 4. Conclusions

After nearly nine decades of investigation, extensive research has consistently demonstrated the profound metabolic benefits of CR across a wide array of model organisms. These benefits span improvements in lifespan and healthspan, primarily through the mitigation of age-associated pathologies such as type 2 diabetes and cardiovascular disease, among others. However, the implications of CR on reproductive physiology, particularly male fertility, remain significantly more nuanced. Male reproductive function, including spermatogenesis, steroidogenesis, and overall testicular homeostasis, is an energetically demanding process tightly regulated by hormonal and metabolic signals. Under conditions of caloric deficit, the organism adapts by reallocating energy toward essential survival pathways, often at the expense of reproductive capacity. This shift, while advantageous for promoting metabolic efficiency and enhancing stress resistance, such as improved insulin sensitivity and reduced oxidative damage, may result in suppressed HPG axis activity, diminished testosterone levels, reduced sperm output, and compromised testicular integrity.

Accordingly, emerging data suggest that the degree and duration of CR play a critical role in determining its reproductive outcomes. Moderate CR (approximately 20 to 30% below ad libitum intake) appears to strike a physiological balance, offering robust metabolic improvements while minimizing the risk of endocrine disruption, which increases with a higher CR percentage. Excessive restriction, on the other hand, may lead to hypogonadism and impaired spermatogenesis, among other effects, particularly in prolonged regimens or during developmental windows. Additionally, as with any other dietary regimen, the recommended CR is highly variable depending on the individual, making it difficult to establish standard recommendations that may apply to the general population.

**Figure 5:**
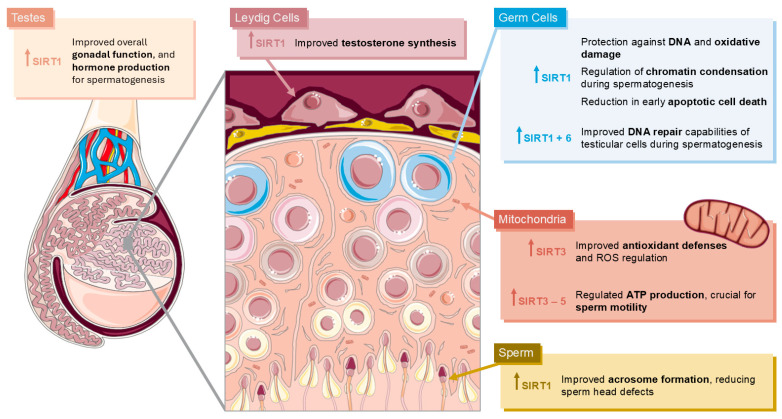
Effects of caloric restriction (CR) on sirtuin (SIRT) regulation, and subsequent impact on male fertility and testicular function. CR is able to impact a variety of SIRTs, which can then modulate several aspects of the male reproductive system. In the testes, the increase in SIRT1 can improve overall gonadal function by ensuring adequate hormone production for spermatogenesis, due to its regulation of the hypothalamic–pituitary–gonadal axis. In Leydig cells, CR increases SIRT1, which improves testosterone synthesis, supporting overall testicular health. In germ cells, the increase in SIRT1 protects germ cells against oxidative stress and DNA damage, facilitates proper histone-to-protamine transition and chromatin remodeling regulating chromatin condensation during spermatogenesis, and suppresses p53, reducing early apoptotic cell death and preserving cell survival. Additionally, SIRT1 and SIRT6 improve the overall DNA repair capabilities of testicular cells, enhancing DNA repair during spermatogenesis. In this way, CR contributes to enhancing the maturing process and integrity of germ cells. In the mitochondria, the increase in SIRT1 activity outside the cells activates the PGC-1α pathway and upregulates SIRT3, which contributes to antioxidant defenses and ROS regulation. Additionally, SIRT3, together with other mitochondrial SIRTs (SIRT4 and 5), regulates ATP production, which is crucial for sperm motility and overall energy metabolism in germ cells. Lastly, in sperm itself, CR upregulates SIRT1, which contributes to acrosome formation and reduces the occurrence of sperm head defects. Abbreviations: ATP, adenosine triphosphate; DNA, Deoxyribonucleic Acid; ROS, reactive oxygen species; SIRT, sirtuin. This figure was drawn using images from Servier Medical Art. Servier Medical Art by Servieris licensed under a Creative Commons Attribution 4.0 Unported License (CC BY) (https://smart.servier.com/citation-sharing/).

Central to these adaptive processes are the SIRTs, which act as metabolic sensors and epigenetic regulators. Sirtuins orchestrate a multitude of cellular programs, including mitochondrial biogenesis, oxidative stress response, lipid and glucose metabolism, and inflammation modulation. The alterations in the male reproductive tract regarding SIRT changes are summarized in Figure 5. Notably, SIRT1, SIRT3, and SIRT6 have been implicated in key testicular functions, such as steroid hormone biosynthesis, protection against germ cell apoptosis, maintenance of Sertoli and Leydig cell viability, and regulation of the blood–testis barrier. Under CR, studies have shown that the upregulation of SIRT1 is able to improve testosterone synthesis in Leydig cells and contribute towards acrosome formation in sperm, reducing sperm head defects. Additionally, in germ cells, SIRT1 offers protection against oxidative damage and, together with SIRT6, enhances DNA repair during spermatogenesis, preserving cell survival. On the other hand, SIRT3 is upregulated in mitochondria under CR conditions, contributing towards antioxidant defenses, as well as regulating ATP production, together with the other mitochondrial SIRTs, which is crucial for sperm motility. However, the role of sirtuins in mediating CR-induced effects on male fertility remains incomplete, particularly lacking regarding evidence in humans. Conflicting findings across animal models underscore the complexity of these interactions: while some studies report enhanced testosterone synthesis, increased sperm quality, and delayed reproductive aging, others reveal heightened sperm morphological abnormalities, disrupted hormonal signaling, and impaired reproductive organ development under similar CR paradigms.

Given the rising prevalence of male infertility, often of idiopathic origin, there is an urgent need for mechanistically grounded and translationally relevant research to elucidate how dietary interventions such as CR modulate reproductive capacity. It is our hope that future studies focus on dose–response relationships, age- and tissue-specific sirtuin activity, and the interaction between CR, sirtuins, and the HPG axis, as well as environmental factors such as pollutants, that may offer clarity regarding unknown infertility causes. It is imperative that the scientific discourse on CR continues to evolve beyond its association with longevity trends, encompassing its implications for fertility and endocrine health. Ultimately, refining our understanding of the metabolic–reproductive axis may pave the way for innovative interventions to preserve both systemic health and reproductive potential in aging males.

## Data Availability

No new data were created or analyzed in this study.

## Abbreviations

The following abbreviations are used in this manuscript:

AMPK: AMP-Activated Protein Kinase; AROS: Active Regulator of SIRT1; ATP: Adenosine Triphosphate; BDNF, Brain-derived Neurotrophic Factor; BPA, Bisphenol A; cAMP: Cyclic Adenosine Monophosphate; Cd, Cadmium; CR: Caloric Restriction; CREB: cAMP-Response Element-Binding Protein; CRTC: CREB-Regulated Transcription Co-Activator; CUL: Cullin; CVDs: Cardiovascular Diseases; DBC1: Deleted in Breast Cancer 1; DCAF: DDB1-CUL4-Associated Factor; DDB: Damage-Specific DNA Binding Protein; DEHP, Di(2-ethylhexyl) Phthalate; DNA: Deoxyribonucleic Acid; ETC: Electron Transport Chain; FOXO: Forkhead Box O; FSH: Follicle-Stimulating Hormone; G6P: Glucose-6-Phosphatase; GABP1: GA-Binding Protein; GAPDH: Glyceraldehyde-3-Phosphate Dehydrogenase; GCN5: General Control Non-Repressed Protein; GDH: Glutamate Dehydrogenase; GLUT: Glucose Transporter; GLP-1: Glucagon-like Peptide-1; GnRH: Gonadotropin-Releasing Hormone; RHOX: Reproductive Homeobox; H3K9: Histone H3 Lysine 9; HAAO: 3-Hydroxyanthranilate 3,4-Dioxygenase; HATs: Histone Acetyltransferases; HDAC: Histone Deacetylase HDAC; HDL: High-density Lipoprotein; HIC: Hypermethylated in Cancer 1 Protein; HIF-1α: Hypoxia-Inducible Factor 1α; HMGCS: 3-Hydroxy-3-Methylglutaryl-CoA Synthase; HPG: Hypothalamic–Pituitary–Gonadal; HuR: protein Hu antigen R; IGF: Insulin-Like Growth Factor; KMO: Kynurenine 3-Monooxygenase; LDH: Lactate dehydrogenase; LDL: Low-density Lipoprotein; LH: Luteinizing Hormone; LXR: Liver X Receptor; mTOR: Mammalian Target of Rapamycin; miRNA: MicroRNA; NA: Nicotinic Acid; NAAD: Nicotinic Acid Adenine Dinucleotide; NAD+: Nicotinamide Adenine Dinucleotide; NADSYN: Glutamine-Dependent NAD(+) Synthetase; NAM: Nicotinamide; NAMN: Nicotinic Acid Mononucleotide; NAMPT: Nicotinamide Phosphoribosyltransferase; NAPRT: Nicotinate Phosphoribosyltransferase; NCoR: Nuclear Receptor Co-Repressors; NFkB: Nuclear Factor kB; NMN: Nicotinamide Mononucleotide; NMNAT: Nicotinamide Mononucleotide Adenylyltransferases; NMRK: Nicotinamide Riboside Kinase; NR: Nicotinamide Riboside; OAT: Oligoasthenoteratozoospermia; PARP: Poly(ADP-ribose) Polymerase; PEPCK-C: Phosphoenolpyruvate Carboxykinase; PGAM-1: Phosphoglycerate Mutase-1; PGC-1α: Peroxisome Proliferator-Activated Receptor Gamma Coactivator-1 Alpha; PM_2.5_, Particulate matter with a diameter of 2.5 μm; PPAR: Peroxisome Proliferator-Activated Receptor; PPARγ: Peroxisome Proliferator-Activated Receptor Gamma; PTP1B: Tyrosine Phosphatase 1B Gene; QAPRT: Quinolinate Phosphoribosyltransferase; ROS: Reactive Oxygen Species; SIRTs: Sirtuins; SMRT: Retinoic Acid and Thyroid Hormone Receptor; SREBP: Sterol Regulatory Element-Binding Protein; TDO: Tryptophan 2,3-Dioxygenase; TR: Testicular Receptor; UCP: Uncoupling Protein.

## References

[1] Bales C.W., Kraus W.E. (2013). Caloric Restriction. J. Cardiopulm. Rehabil. Prev..

[2] Anderson R.M., Shanmuganayagam D., Weindruch R. (2009). Caloric Restriction and Aging: Studies in Mice and Monkeys. Toxicol. Pathol..

[3] Brownlow B.S., Park C.R., Schwartz R.S., Woods S.C. (1993). Effect of meal pattern during food restriction on body weight loss and recovery after refeeding. Physiol. Behav..

[4] Velingkaar N., Mezhnina V., Poe A., Makwana K., Tulsian R., Kondratov R.V. (2020). Reduced caloric intake and periodic fasting independently contribute to metabolic effects of caloric restriction. Aging Cell.

[5] Redman L.M., Heilbronn L.K., Martin C.K., De Jonge L., Williamson D.A., Delany J.P., Ravussin E. (2009). Metabolic and Behavioral Compensations in Response to Caloric Restriction: Implications for the Maintenance of Weight Loss. PLoS ONE.

[6] Redman L.M., Ravussin E. (2011). Caloric Restriction in Humans: Impact on Physiological, Psychological, and Behavioral Outcomes. Antioxid. Redox Signal..

[7] Flanagan E.W., Most J., Mey J.T., Redman L.M. (2020). Calorie Restriction and Aging in Humans. Annu. Rev. Nutr..

[8] Fusco S., Pani G. (2013). Brain response to calorie restriction. Cell. Mol. Life Sci..

[9] de Souza A.M.A., Ecelbarger C.M., Sandberg K. (2021). Caloric Restriction and Cardiovascular Health: The Good, the Bad, and the Renin-Angiotensin System. Physiology.

[10] Kökten T., Hansmannel F., Ndiaye N.C., Heba A.C., Quilliot D., Dreumont N., Arnone D., Peyrin-Biroulet L. (2021). Calorie Restriction as a New Treatment of Inflammatory Diseases. Adv. Nutr..

[11] Dirks A.J., Leeuwenburgh C. (2006). Tumor necrosis factor α signaling in skeletal muscle: Effects of age and caloric restriction. J. Nutr. Biochem..

[12] Suchacki K.J., Thomas B.J., Ikushima Y.M., Chen K.C., Fyfe C., Tavares A.A.S., Sulston R.J., Lovdel A., Woodward H.J., Han X. (2023). The effects of caloric restriction on adipose tissue and metabolic health are sex- and age-dependent. eLife.

[13] Sbierski-Kind J., Grenkowitz S., Schlickeiser S., Sandforth A., Friedrich M., Kunkel D., Glauben R., Brachs S., Mai K., Thürmer A. (2022). Effects of caloric restriction on the gut microbiome are linked with immune senescence. Microbiome.

[14] Dorling J.L., Ravussin E., Redman L.M., Bhapkar M., Huffman K.M., Racette S.B., Das S.K., Apolzan J.W., Kraus W.E., Höchsmann C. (2021). Effect of 2 years of calorie restriction on liver biomarkers: Results from the CALERIE phase 2 randomized controlled trial. Eur. J. Nutr..

[15] Leslie S.W., Soon-Sutton T.L., Khan M.A.B. (2025). Male Infertility. StatPearls.

[16] Levine H., Jørgensen N., Martino-Andrade A., Mendiola J., Weksler-Derri D., Mindlis I., Pinotti R., Swan S.H. (2017). Temporal trends in sperm count: A systematic review and meta-regression analysis. Hum. Reprod. Update.

[17] Danielewicz A., Przybyłowicz K.E., Przybyłowicz M. (2018). Dietary Patterns and Poor Semen Quality Risk in Men: A Cross-Sectional Study. Nutrients.

[18] Herzig S., Shaw R.J. (2018). AMPK: Guardian of metabolism and mitochondrial homeostasis. Nat. Rev. Mol. Cell Biol..

[19] Haeusler R.A., McGraw T.E., Accili D. (2018). Biochemical and cellular properties of insulin receptor signalling. Nat. Rev. Mol. Cell Biol..

[20] Moreira B.P., Oliveira P.F., Alves M.G. (2019). Molecular Mechanisms Controlled by mTOR in Male Reproductive System. Int. J. Mol. Sci..

[21] Ye X., Li M., Hou T., Gao T., Zhu W.-G., Yang Y. (2017). Sirtuins in glucose and lipid metabolism. Oncotarget.

[22] Khoury G.A., Baliban R.C., Floudas C.A. (2011). Proteome-wide post-translational modification statistics: Frequency analysis and curation of the swiss-prot database. Sci. Rep..

[23] Yang X.J. (2004). The diverse superfamily of lysine acetyltransferases and their roles in leukemia and other diseases. Nucleic Acids Res..

[24] Shahbazian M.D., Grunstein M. (2007). Functions of Site-Specific Histone Acetylation and Deacetylation. Annu. Rev. Biochem..

[25] Clayton A.L., Hazzalin C.A., Mahadevan L.C. (2006). Enhanced Histone Acetylation and Transcription: A Dynamic Perspective. Mol. Cell.

[26] Zentner G.E., Henikoff S. (2013). Regulation of nucleosome dynamics by histone modifications. Nat. Struct. Mol. Biol..

[27] Gallinari P., Marco S.D., Jones P., Pallaoro M., Steinkühler C. (2007). HDACs, histone deacetylation and gene transcription: From molecular biology to cancer therapeutics. Cell Res..

[28] Klar A.J., Fogel S., Macleod K. (1979). MAR1-a Regulator of the HMa and HMalpha Loci in *Saccharomyces cerevisiae*. Genetics.

[29] Hopper A.K., Hall B.D. (1975). Mutation of a heterothallic strain to homothallism. Genetics.

[30] Rine J., Strathern J.N., Hicks J.B., Herskowitz I. (1979). A suppressor of mating-type locus mutations in *Saccharomyces cerevisiae*: Evidence for and identification of cryptic mating-type loci. Genetics.

[31] Mendes K.L., Lelis D.d.F., Santos S.H.S. (2017). Nuclear sirtuins and inflammatory signaling pathways. Cytokine Growth Factor Rev..

[32] Singh C.K., Chhabra G., Ndiaye M.A., Garcia-Peterson L.M., Mack N.J., Ahmad N. (2018). The Role of Sirtuins in Antioxidant and Redox Signaling. Antioxid. Redox Signal..

[33] O’Callaghan C., Vassilopoulos A. (2017). Sirtuins at the crossroads of stemness, aging, and cancer. Aging Cell.

[34] McBurney M.W., Yang X., Jardine K., Hixon M., Boekelheide K., Webb J.R., Lansdorp P.M., Lemieux M. (2003). The mammalian SIR2alpha protein has a role in embryogenesis and gametogenesis. Mol. Cell. Biol..

[35] Guan X., Lin P., Knoll E., Chakrabarti R. (2014). Mechanism of inhibition of the human sirtuin enzyme SIRT3 by nicotinamide: Computational and experimental studies. PLoS ONE.

[36] Tong L., Denu J.M. (2010). Function and metabolism of sirtuin metabolite O-acetyl-ADP-ribose. Biochim. Biophys. Acta.

[37] Ford E., Voit R., Liszt G., Magin C., Grummt I., Guarente L. (2006). Mammalian Sir2 homolog SIRT7 is an activator of RNA polymerase I transcription. Genes Dev..

[38] Tanno M., Sakamoto J., Miura T., Shimamoto K., Horio Y. (2007). Nucleocytoplasmic shuttling of the NAD^+^-dependent histone deacetylase SIRT1. J. Biol. Chem..

[39] Osborne B., Bentley N.L., Montgomery M.K., Turner N. (2016). The role of mitochondrial sirtuins in health and disease. Free Radic. Biol. Med..

[40] Vaquero A., Scher M.B., Lee D.H., Sutton A., Cheng H.L., Alt F.W., Serrano L., Sternglanz R., Reinberg D. (2006). SirT2 is a histone deacetylase with preference for histone H4 Lys 16 during mitosis. Genes Dev..

[41] Ardestani P.M., Liang F. (2012). Sub-cellular localization, expression and functions of Sirt6 during the cell cycle in HeLa cells. Nucleus.

[42] Budayeva H.G., Cristea I.M. (2016). Human Sirtuin 2 Localization, Transient Interactions, and Impact on the Proteome Point to Its Role in Intracellular Trafficking. Mol. Cell. Proteom..

[43] Bai W., Zhang X. (2016). Nucleus or cytoplasm? The mysterious case of SIRT1’s subcellular localization. Cell Cycle.

[44] Kaeberlein M., McVey M., Guarente L. (1999). The SIR2/3/4 complex and SIR2 alone promote longevity in *Saccharomyces cerevisiae* by two different mechanisms. Genes Dev..

[45] Noriega L.G., Feige J.N., Canto C., Yamamoto H., Yu J., Herman M.A., Mataki C., Kahn B.B., Auwerx J. (2011). CREB and ChREBP oppositely regulate SIRT1 expression in response to energy availability. Eur. Mol. Biol. Organ. Rep..

[46] Hayashida S., Arimoto A., Kuramoto Y., Kozako T., Honda S., Shimeno H., Soeda S. (2010). Fasting promotes the expression of SIRT1, an NAD^+^-dependent protein deacetylase, via activation of PPARalpha in mice. Mol. Cell. Biochem..

[47] Nemoto S., Fergusson M.M., Finkel T. (2004). Nutrient availability regulates SIRT1 through a forkhead-dependent pathway. Science.

[48] Bai P., Canto C., Brunyánszki A., Huber A., Szántó M., Cen Y., Yamamoto H., Houten S.M., Kiss B., Oudart H. (2011). PARP-2 regulates SIRT1 expression and whole-body energy expenditure. Cell Metab..

[49] Chen W.Y., Wang D.H., Yen R.C., Luo J., Gu W., Baylin S.B. (2005). Tumor suppressor HIC1 directly regulates SIRT1 to modulate p53-dependent DNA-damage responses. Cell.

[50] Pan S., Cui Y., Fu Z., Zhang L., Xing H. (2019). MicroRNA-128 is involved in dexamethasone-induced lipid accumulation via repressing SIRT1 expression in cultured pig preadipocytes. J. Steroid Biochem. Mol. Biol..

[51] Tian Z., Jiang H., Liu Y., Huang Y., Xiong X., Wu H., Dai X. (2016). MicroRNA-133b inhibits hepatocellular carcinoma cell progression by targeting Sirt1. Exp. Cell Res..

[52] Yang Y., Liu Y., Xue J., Yang Z., Shi Y., Shi Y., Lou G., Wu S., Qi J., Liu W. (2017). MicroRNA-141 Targets Sirt1 and Inhibits Autophagy to Reduce HBV Replication. Cell. Physiol. Biochem..

[53] Abdelmohsen K., Pullmann R., Lal A., Kim H.H., Galban S., Yang X., Blethrow J.D., Walker M., Shubert J., Gillespie D.A. (2007). Phosphorylation of HuR by Chk2 regulates SIRT1 expression. Mol. Cell.

[54] Kokkola T., Suuronen T., Molnár F., Määttä J., Salminen A., Jarho E.M., Lahtela-Kakkonen M. (2014). AROS has a context-dependent effect on SIRT1. Fed. Eur. Biochem. Soc. Lett..

[55] Yuan J., Luo K., Liu T., Lou Z. (2012). Regulation of SIRT1 activity by genotoxic stress. Genes Dev..

[56] Tan F., Dong W., Lei X., Liu X., Li Q., Kang L., Zhao S., Zhang C. (2018). Attenuated SUMOylation of sirtuin 1 in premature neonates with bronchopulmonary dysplasia. Mol. Med. Rep..

[57] Han X., Niu J., Zhao Y., Kong Q., Tong T., Han L. (2016). HDAC4 stabilizes SIRT1 via sumoylation SIRT1 to delay cellular senescence. Clin. Exp. Pharmacol. Physiol..

[58] Wang W., Li F., Xu Y., Wei J., Zhang Y., Yang H., Gao B., Yu G., Fang D. (2018). JAK1-mediated Sirt1 phosphorylation functions as a negative feedback of the JAK1-STAT3 pathway. J. Biol. Chem..

[59] Choi S.E., Kwon S., Seok S., Xiao Z., Lee K.W., Kang Y., Li X., Shinoda K., Kajimura S., Kemper B. (2017). Obesity-Linked Phosphorylation of SIRT1 by Casein Kinase 2 Inhibits Its Nuclear Localization and Promotes Fatty Liver. Mol. Cell. Biol..

[60] Tulino R., Benjamin A.C., Jolinon N., Smith D.L., Chini E.N., Carnemolla A., Bates G.P. (2016). SIRT1 Activity Is Linked to Its Brain Region-Specific Phosphorylation and Is Impaired in Huntington’s Disease Mice. PLoS ONE.

[61] Cantó C., Menzies K.J., Auwerx J. (2015). NAD^+^ Metabolism and the Control of Energy Homeostasis: A Balancing Act between Mitochondria and the Nucleus. Cell Metab..

[62] Yang Y., Sauve A.A. (2016). NAD^+^ metabolism: Bioenergetics, signaling and manipulation for therapy. Biochim. Biophys. Acta.

[63] Ohashi K., Kawai S., Murata K. (2013). Secretion of quinolinic acid, an intermediate in the kynurenine pathway, for utilization in NAD+ biosynthesis in the yeast *Saccharomyces cerevisiae*. Eukaryot. Cell.

[64] Srivastava S. (2016). Emerging therapeutic roles for NAD^+^ metabolism in mitochondrial and age-related disorders. Clin. Transl. Med..

[65] Nikiforov A., Kulikova V., Ziegler M. (2015). The human NAD metabolome: Functions, metabolism and compartmentalization. Crit. Rev. Biochem. Mol. Biol..

[66] Jokinen R., Pirnes-Karhu S., Pietiläinen K.H., Pirinen E. (2017). Adipose tissue NAD^+^-homeostasis, sirtuins and poly(ADP-ribose) polymerases -important players in mitochondrial metabolism and metabolic health. Redox Biol.

[67] Bai P., Cantó C., Oudart H., Brunyánszki A., Cen Y., Thomas C., Yamamoto H., Huber A., Kiss B., Houtkooper R.H. (2011). PARP-1 inhibition increases mitochondrial metabolism through SIRT1 activation. Cell Metab..

[68] Jęśko H., Strosznajder R.P. (2016). Sirtuins and their interactions with transcription factors and poly(ADP-ribose) polymerases. Folia Neuropathol..

[69] Gupte R., Liu Z., Kraus W.L. (2017). PARPs and ADP-ribosylation: Recent advances linking molecular functions to biological outcomes. Genes Dev..

[70] Nakrani M.N., Wineland R.H., Anjum F. (2023). Physiology, Glucose Metabolism. StatPearls.

[71] Noguchi R., Kubota H., Yugi K., Toyoshima Y., Komori Y., Soga T., Kuroda S. (2013). The selective control of glycolysis, gluconeogenesis and glycogenesis by temporal insulin patterns. Mol. Syst. Biol..

[72] Zhong L., D’Urso A., Toiber D., Sebastian C., Henry R.E., Vadysirisack D.D., Guimaraes A., Marinelli B., Wikstrom J.D., Nir T. (2010). The Histone Deacetylase Sirt6 Regulates Glucose Homeostasis via Hif1α. Cell.

[73] Rodgers J.T., Lerin C., Haas W., Gygi S.P., Spiegelman B.M., Puigserver P. (2005). Nutrient control of glucose homeostasis through a complex of PGC-1α and SIRT1. Nature.

[74] Kane A.E., Sinclair D.A. (2018). Sirtuins and NAD^+^ in the Development and Treatment of Metabolic and Cardiovascular Diseases. Circ. Res..

[75] Kitamura Y.I., Kitamura T., Kruse J.-P., Raum J.C., Stein R., Gu W., Accili D. (2005). FoxO1 protects against pancreatic β cell failure through NeuroD and MafA induction. Cell Metab..

[76] Moynihan K.A., Grimm A.A., Plueger M.M., Bernal-Mizrachi E., Ford E., Cras-Méneur C., Permutt M.A., Imai S.-I. (2005). Increased dosage of mammalian Sir2 in pancreatic β cells enhances glucose-stimulated insulin secretion in mice. Cell Metab..

[77] Bordone L., Motta M.C., Picard F., Robinson A., Jhala U.S., Apfeld J., McDonagh T., Lemieux M., McBurney M., Szilvasi A. (2005). Sirt1 Regulates Insulin Secretion by Repressing UCP2 in Pancreatic β Cells. PLoS Biol..

[78] Nemoto S., Fergusson M.M., Finkel T. (2005). SIRT1 Functionally Interacts with the Metabolic Regulator and Transcriptional Coactivator PGC-1α. J. Biol. Chem..

[79] Zhang H.-H., Ma X.-J., Wu L.-N., Zhao Y.-Y., Zhang P.-Y., Zhang Y.-H., Shao M.-W., Liu F., Li F., Qin G.-J. (2015). SIRT1 attenuates high glucose-induced insulin resistance via reducing mitochondrial dysfunction in skeletal muscle cells. Exp. Biol. Med..

[80] Hu X., Chi L., Zhang W., Bai T., Zhao W., Feng Z., Tian H. (2015). Down-regulation of the miR-543 alleviates insulin resistance through targeting the SIRT1. Biochem. Biophys. Res. Commun..

[81] Rodgers J.T., Puigserver P. (2007). Fasting-dependent glucose and lipid metabolic response through hepatic sirtuin 1. Proc. Natl. Acad. Sci. USA.

[82] Koo S.H., Satoh H., Herzig S., Lee C.H., Hedrick S., Kulkarni R., Evans R.M., Olefsky J., Montminy M. (2004). PGC-1 promotes insulin resistance in liver through PPAR-alpha-dependent induction of TRB-3. Nat. Med..

[83] Zabolotny J.M., Kim Y.-B. (2007). Silencing Insulin Resistance through SIRT1. Cell Metab..

[84] Jing E., Emanuelli B., Hirschey M.D., Boucher J., Lee K.Y., Lombard D., Verdin E.M., Kahn C.R. (2011). Sirtuin-3 (Sirt3) regulates skeletal muscle metabolism and insulin signaling via altered mitochondrial oxidation and reactive oxygen species production. Proc. Natl. Acad. Sci. USA.

[85] Haigis M.C., Mostoslavsky R., Haigis K.M., Fahie K., Christodoulou D.C., Murphy A.J., Yancopoulos G.D., Karow M., Blander G., Wolberger C. (2006). SIRT4 Inhibits Glutamate Dehydrogenase and Opposes the Effects of Calorie Restriction in Pancreatic β Cells. Cell.

[86] Zaganjor E., Vyas S., Haigis M.C. (2017). SIRT4 Is a Regulator of Insulin Secretion. Cell Chem. Biol..

[87] Oonk R.B., Grootegoed J.A. (1987). Identification of insulin receptors on rat Sertoli cells. Mol. Cell. Endocrinol..

[88] Ahn S.W., Gang G.-T., Kim Y.D., Ahn R.-S., Harris R.A., Lee C.-H., Choi H.-S. (2013). Insulin Directly Regulates Steroidogenesis via Induction of the Orphan Nuclear Receptor DAX-1 in Testicular Leydig Cells*. J. Biol. Chem..

[89] Ballester J., Muñoz M.C., Domínguez J., Rigau T., Guinovart J.J., Rodríguez-Gil J.E. (2004). Insulin-dependent diabetes affects testicular function by FSH- and LH-linked mechanisms. J. Androl..

[90] Ma M.C., Chiu T.J., Lu H.I., Huang W.T., Lo C.M., Tien W.Y., Lan Y.C., Chen Y.Y., Chen C.H., Li S.H. (2018). SIRT1 overexpression is an independent prognosticator for patients with esophageal squamous cell carcinoma. J. Cardiothorac. Surg..

[91] Liu Y., Dentin R., Chen D., Hedrick S., Ravnskjaer K., Schenk S., Milne J., Meyers D.J., Cole P., Yates J. (2008). A fasting inducible switch modulates gluconeogenesis via activator/coactivator exchange. Nature.

[92] Wang F., Tong Q. (2009). SIRT2 suppresses adipocyte differentiation by deacetylating FOXO1 and enhancing FOXO1’s repressive interaction with PPARgamma. Mol. Biol. Cell.

[93] Hallows W.C., Yu W., Denu J.M. (2012). Regulation of glycolytic enzyme phosphoglycerate mutase-1 by Sirt1 protein-mediated deacetylation. J. Biol. Chem..

[94] Nogueiras R., Habegger K.M., Chaudhary N., Finan B., Banks A.S., Dietrich M.O., Horvath T.L., Sinclair D.A., Pfluger P.T., Tschöp M.H. (2012). Sirtuin 1 and sirtuin 3: Physiological modulators of metabolism. Physiol. Rev..

[95] Jiang W., Wang S., Xiao M., Lin Y., Zhou L., Lei Q., Xiong Y., Guan K.L., Zhao S. (2011). Acetylation regulates gluconeogenesis by promoting PEPCK1 degradation via recruiting the UBR5 ubiquitin ligase. Mol. Cell.

[96] Dowell P., Otto T.C., Adi S., Lane M.D. (2003). Convergence of peroxisome proliferator-activated receptor gamma and Foxo1 signaling pathways. J. Biol. Chem..

[97] Lang A., Grether-Beck S., Singh M., Kuck F., Jakob S., Kefalas A., Altinoluk-Hambüchen S., Graffmann N., Schneider M., Lindecke A. (2016). MicroRNA-15b regulates mitochondrial ROS production and the senescence-associated secretory phenotype through sirtuin 4/SIRT4. Aging.

[98] Ahuja N., Schwer B., Carobbio S., Waltregny D., North B.J., Castronovo V., Maechler P., Verdin E. (2007). Regulation of insulin secretion by SIRT4, a mitochondrial ADP-ribosyltransferase. J. Biol. Chem..

[99] Gertz M., Steegborn C. (2010). Function and regulation of the mitochondrial sirtuin isoform Sirt5 in Mammalia. Biochim. Biophys. Acta.

[100] Yang X., Liu B., Zhu W., Luo J. (2015). SIRT5, functions in cellular metabolism with a multiple enzymatic activities. Sci. China Life Sci..

[101] Nishida Y., Rardin M.J., Carrico C., He W., Sahu A.K., Gut P., Najjar R., Fitch M., Hellerstein M., Gibson B.W. (2015). SIRT5 Regulates both Cytosolic and Mitochondrial Protein Malonylation with Glycolysis as a Major Target. Mol. Cell.

[102] Rardin M.J., He W., Nishida Y., Newman J.C., Carrico C., Danielson S.R., Guo A., Gut P., Sahu A.K., Li B. (2013). SIRT5 regulates the mitochondrial lysine succinylome and metabolic networks. Cell Metab..

[103] Xiao C., Kim H.S., Lahusen T., Wang R.H., Xu X., Gavrilova O., Jou W., Gius D., Deng C.X. (2010). SIRT6 deficiency results in severe hypoglycemia by enhancing both basal and insulin-stimulated glucose uptake in mice. J. Biol. Chem..

[104] Parenti M.D., Grozio A., Bauer I., Galeno L., Damonte P., Millo E., Sociali G., Franceschi C., Ballestrero A., Bruzzone S. (2014). Discovery of novel and selective SIRT6 inhibitors. J. Med. Chem..

[105] Sebastián C., Zwaans B.M., Silberman D.M., Gymrek M., Goren A., Zhong L., Ram O., Truelove J., Guimaraes A.R., Toiber D. (2012). The histone deacetylase SIRT6 is a tumor suppressor that controls cancer metabolism. Cell.

[106] Wu M., Seto E., Zhang J. (2015). E2F1 enhances glycolysis through suppressing Sirt6 transcription in cancer cells. Oncotarget.

[107] Kugel S., Mostoslavsky R. (2014). Chromatin and beyond: The multitasking roles for SIRT6. Trends Biochem. Sci..

[108] Picard F., Kurtev M., Chung N., Topark-Ngarm A., Senawong T., Machado De Oliveira R., Leid M., McBurney M.W., Guarente L. (2004). Sirt1 promotes fat mobilization in white adipocytes by repressing PPAR-gamma. Nature.

[109] Simmons G.E., Pruitt W.M., Pruitt K. (2015). Diverse roles of SIRT1 in cancer biology and lipid metabolism. Int. J. Mol. Sci..

[110] Rodgers J.T., Lerin C., Gerhart-Hines Z., Puigserver P. (2008). Metabolic adaptations through the PGC-1 alpha and SIRT1 pathways. Fed. Eur. Biochem. Soc. Lett..

[111] Frescas D., Valenti L., Accili D. (2005). Nuclear trapping of the forkhead transcription factor FoxO1 via Sirt-dependent deacetylation promotes expression of glucogenetic genes. J. Biol. Chem..

[112] Feige J.N., Auwerx J. (2007). DisSIRTing on LXR and cholesterol metabolism. Cell Metab..

[113] Zelcer N., Tontonoz P. (2006). Liver X receptors as integrators of metabolic and inflammatory signaling. J. Clin. Investig..

[114] Nasrin N., Wu X., Fortier E., Feng Y., Bare O.C., Chen S., Ren X., Wu Z., Streeper R.S., Bordone L. (2010). SIRT4 regulates fatty acid oxidation and mitochondrial gene expression in liver and muscle cells. J. Biol. Chem..

[115] Laurent G., de Boer V.C., Finley L.W., Sweeney M., Lu H., Schug T.T., Cen Y., Jeong S.M., Li X., Sauve A.A. (2013). SIRT4 represses peroxisome proliferator-activated receptor α activity to suppress hepatic fat oxidation. Mol. Cell. Biol..

[116] Kanfi Y., Peshti V., Gil R., Naiman S., Nahum L., Levin E., Kronfeld-Schor N., Cohen H.Y. (2010). SIRT6 protects against pathological damage caused by diet-induced obesity. Aging Cell.

[117] Tao R., Xiong X., DePinho R.A., Deng C.X., Dong X.C. (2013). Hepatic SREBP-2 and cholesterol biosynthesis are regulated by FoxO3 and Sirt6. J. Lipid Res..

[118] Kim H.S., Xiao C., Wang R.H., Lahusen T., Xu X., Vassilopoulos A., Vazquez-Ortiz G., Jeong W.I., Park O., Ki S.H. (2010). Hepatic-specific disruption of SIRT6 in mice results in fatty liver formation due to enhanced glycolysis and triglyceride synthesis. Cell Metab..

[119] Shin J., He M., Liu Y., Paredes S., Villanova L., Brown K., Qiu X., Nabavi N., Mohrin M., Wojnoonski K. (2013). SIRT7 represses Myc activity to suppress ER stress and prevent fatty liver disease. Cell Rep..

[120] Ryu D., Jo Y.S., Lo Sasso G., Stein S., Zhang H., Perino A., Lee J.U., Zeviani M., Romand R., Hottiger M.O. (2014). A SIRT7-dependent acetylation switch of GABPβ1 controls mitochondrial function. Cell Metab..

[121] Yoshizawa T., Karim M.F., Sato Y., Senokuchi T., Miyata K., Fukuda T., Go C., Tasaki M., Uchimura K., Kadomatsu T. (2014). SIRT7 controls hepatic lipid metabolism by regulating the ubiquitin-proteasome pathway. Cell Metab..

[122] Hubbi M.E., Hu H., Kshitiz, Gilkes D.M., Semenza G.L. (2013). Sirtuin-7 inhibits the activity of hypoxia-inducible factors. J. Biol. Chem..

[123] Cardoso A.M., Alves M.G., Sousa A.C., Jarak I., Carvalho R.A., Oliveira P.F., Cavaco J.E., Rato L. (2018). The effects of the obesogen tributyltin on the metabolism of Sertoli cells cultured ex vivo. Arch. Toxicol..

[124] Martins A.D., Sá R., Monteiro M.P., Barros A., Sousa M., Carvalho R.A., Silva B.M., Oliveira P.F., Alves M.G. (2016). Ghrelin acts as energy status sensor of male reproduction by modulating Sertoli cells glycolytic metabolism and mitochondrial bioenergetics. Mol. Cell. Endocrinol..

[125] Martins A.D., Moreira A.C., Sá R., Monteiro M.P., Sousa M., Carvalho R.A., Silva B.M., Oliveira P.F., Alves M.G. (2015). Leptin modulates human Sertoli cells acetate production and glycolytic profile: A novel mechanism of obesity-induced male infertility?. Biochim. Biophys. Acta.

[126] Martins A.D., Monteiro M.P., Silva B.M., Barros A., Sousa M., Carvalho R.A., Oliveira P.F., Alves M.G. (2019). Metabolic dynamics of human Sertoli cells are differentially modulated by physiological and pharmacological concentrations of GLP-1. Toxicol. Appl. Pharmacol..

[127] Moreira B.P., Silva J.F., Jarak I., de Lourdes Pereira M., Oliveira P.F., Alves M.G. (2020). Technical-grade chlordane compromises rat Sertoli cells proliferation, viability and metabolic activity. Toxicol. Vitr..

[128] Oliveira P.F., Sousa M., Silva B.M., Monteiro M.P., Alves M.G. (2017). Obesity, energy balance and spermatogenesis. Reproduction.

[129] Ramaswamy S., Weinbauer G.F. (2014). Endocrine control of spermatogenesis: Role of FSH and LH/ testosterone. Spermatogenesis.

[130] Kolthur-Seetharam U., Teerds K., de Rooij D.G., Wendling O., McBurney M., Sassone-Corsi P., Davidson I. (2009). The histone deacetylase SIRT1 controls male fertility in mice through regulation of hypothalamic-pituitary gonadotropin signaling. Biol. Reprod..

[131] Di Sante G., Wang L., Wang C., Jiao X., Casimiro M.C., Chen K., Pestell T.G., Yaman I., Di Rocco A., Sun X. (2015). Sirt1-deficient mice have hypogonadotropic hypogonadism due to defective GnRH neuronal migration. J. Mol. Endocrinol..

[132] Martins A.D., Jarak I., Morais T., Carvalho R.A., Oliveira P.F., Monteiro M.P., Alves M.G. (2020). Caloric restriction alters the hormonal profile and testicular metabolome, resulting in alterations of sperm head morphology. Am. J. Physiol.-Endocrinol. Metab..

[133] Jesús P.L., Arenas-Ríos E., Ruíz-Ramos M., Flores-Alonso J.C., Mendoza-Núñez V.M., Arrieta-Cruz I., Arteaga-Silva M. (2022). Effect of Chronic Moderate Caloric Restriction on the Reproductive Function in Aged Male Wistar Rats. Nutrients.

[134] Sitzmann B.D., Leone E.H., Mattison J.A., Ingram D.K., Roth G.S., Urbanski H.F., Zelinski M.B., Ottinger M.A. (2010). Effects of moderate calorie restriction on testosterone production and semen characteristics in young rhesus macaques (Macaca mulatta). Biol. Reprod..

[135] Sitzmann B.D., Mattison J.A., Ingram D.K., Roth G.S., Ottinger M.A., Urbanski H.F. (2010). Impact of Moderate Calorie Restriction on the Reproductive Neuroendocrine Axis of Male Rhesus Macaques. Open Longev. Sci..

[136] Zheng X.M., Zhang X.D., Tan L.L., Zhang J., Wang T.T., Ling Q., Wang H., Ouyang K.W., Wang K.W., Chang W. (2024). Sirt1 m6A modification-evoked Leydig cell senescence promotes Cd-induced testosterone decline. Ecotoxicol. Environ. Saf..

[137] Dong W., Zhang K., Liu G., Tan Y., Zou H., Yuan Y., Gu J., Song R., Zhu J., Liu Z. (2021). Puerarin prevents cadmium-induced disorder of testicular lactic acid metabolism in rats by activating 5′ AMP-activated protein kinase (AMPK)/sirtuin 1 (SIRT1) signaling pathway. Environ. Toxicol..

[138] Wang M., Zhu C.-Q., Zeng L., Cheng L., Ma L., Zhang M., Zhang Y.-Z. (2021). Melatonin regulates the cross-talk between autophagy and apoptosis by SIRT3 in testicular Leydig cells. Biochem. Biophys. Res. Commun..

[139] Ye F., Wu L., Li H., Peng X., Xu Y., Li W., Wei Y., Chen F., Zhang J., Liu Q. (2023). SIRT1/PGC-1α is involved in arsenic-induced male reproductive damage through mitochondrial dysfunction, which is blocked by the antioxidative effect of zinc. Environ. Pollut..

[140] Abd-Elhakim Y.M., El Sharkawy N.I., El Bohy K.M., Hassan M.A., Gharib H.S.A., El-Metwally A.E., Arisha A.H., Imam T.S. (2021). Iprodione and/or chlorpyrifos exposure induced testicular toxicity in adult rats by suppression of steroidogenic genes and SIRT1/TERT/PGC-1α pathway. Environ. Sci. Pollut. Res. Int..

[141] Liang Y., Yang Y., Lu C., Cheng Y., Jiang X., Yang B., Li Y., Chen Q., Ao L., Cao J. (2024). Polystyrene nanoplastics exposure triggers spermatogenic cell senescence via the Sirt1/ROS axis. Ecotoxicol. Environ. Saf..

[142] Zheng S., Jiang J., Shu Z., Qiu C., Jiang L., Zhao N., Lin X., Qian Y., Liang B., Qiu L. (2024). Fine particulate matter (PM(2.5)) induces testosterone disruption by triggering ferroptosis through SIRT1/HIF-1alpha signaling pathway in male mice. Free Radic. Biol. Med..

[143] Zhao Y., Li M.Z., Talukder M., Luo Y., Shen Y., Wang H.R., Li J.L. (2020). Effect of mitochondrial quality control on the lycopene antagonizing DEHP-induced mitophagy in spermatogenic cells. Food Funct..

[144] Borra M.T., Smith B.C., Denu J.M. (2005). Mechanism of human SIRT1 activation by resveratrol. J. Biol. Chem..

[145] Gertz M., Nguyen G.T., Fischer F., Suenkel B., Schlicker C., Fränzel B., Tomaschewski J., Aladini F., Becker C., Wolters D. (2012). A molecular mechanism for direct sirtuin activation by resveratrol. PLoS ONE.

[146] Ciccone L., Piragine E., Brogi S., Camodeca C., Fucci R., Calderone V., Nencetti S., Martelli A., Orlandini E. (2022). Resveratrol-like Compounds as SIRT1 Activators. Int. J. Mol. Sci..

[147] Chao S.C., Chen Y.J., Huang K.H., Kuo K.L., Yang T.H., Huang K.Y., Wang C.C., Tang C.H., Yang R.S., Liu S.H. (2017). Induction of sirtuin-1 signaling by resveratrol induces human chondrosarcoma cell apoptosis and exhibits antitumor activity. Sci. Rep..

[148] Bastianetto S., Ménard C., Quirion R. (2015). Neuroprotective action of resveratrol. Biochim. Biophys. Acta.

[149] Walker W.H. (2011). Testosterone signaling and the regulation of spermatogenesis. Spermatogenesis.

[150] Verón G.L., Tissera A.D., Bello R., Beltramone F., Estofan G., Molina R.I., Vazquez-Levin M.H. (2018). Impact of age, clinical conditions, and lifestyle on routine semen parameters and sperm kinematics. Fertil. Steril..

[151] Zhang F.P., Pakarainen T., Zhu F., Poutanen M., Huhtaniemi I. (2004). Molecular characterization of postnatal development of testicular steroidogenesis in luteinizing hormone receptor knockout mice. Endocrinology.

[152] Zhang F.P., Poutanen M., Wilbertz J., Huhtaniemi I. (2001). Normal prenatal but arrested postnatal sexual development of luteinizing hormone receptor knockout (LuRKO) mice. Mol. Endocrinol..

[153] Ma X., Dong Y., Matzuk M.M., Kumar T.R. (2004). Targeted disruption of luteinizing hormone beta-subunit leads to hypogonadism, defects in gonadal steroidogenesis, and infertility. Proc. Natl. Acad. Sci. USA.

[154] Lei Z.M., Mishra S., Zou W., Xu B., Foltz M., Li X., Rao C.V. (2001). Targeted disruption of luteinizing hormone/human chorionic gonadotropin receptor gene. Mol. Endocrinol..

[155] Michishita E., Park J.Y., Burneskis J.M., Barrett J.C., Horikawa I. (2005). Evolutionarily conserved and nonconserved cellular localizations and functions of human SIRT proteins. Mol. Biol. Cell.

[156] Kraus W., Bhapkar M., Huffman K., Pieper C., Das S., Redman L., Villareal D., Rochon J., Roberts S., Ravussin E. (2019). 2 years of calorie restriction and cardiometabolic risk (CALERIE): Exploratory outcomes of a multicentre, phase 2, randomised controlled trial. Lancet Diabetes Endocrinol..

[157] Coussens M., Maresh J.G., Yanagimachi R., Maeda G., Allsopp R. (2008). Sirt1 deficiency attenuates spermatogenesis and germ cell function. PLoS ONE.

[158] Bell E.L., Nagamori I., Williams E.O., Del Rosario A.M., Bryson B.D., Watson N., White F.M., Sassone-Corsi P., Guarente L. (2014). SirT1 is required in the male germ cell for differentiation and fecundity in mice. Development.

[159] Beumer T.L., Roepers-Gajadien H.L., Gademan I.S., van Buul P.P., Gil-Gomez G., Rutgers D.H., de Rooij D.G. (1998). The role of the tumor suppressor p53 in spermatogenesis. Cell Death Differ..

[160] Allemand I., Anglo A., Jeantet A.Y., Cerutti I., May E. (1999). Testicular wild-type p53 expression in transgenic mice induces spermiogenesis alterations ranging from differentiation defects to apoptosis. Oncogene.

[161] Kong X., Wang R., Xue Y., Liu X., Zhang H., Chen Y., Fang F., Chang Y. (2010). Sirtuin 3, a new target of PGC-1alpha, plays an important role in the suppression of ROS and mitochondrial biogenesis. PLoS ONE.

[162] Rato L., Duarte A.I., Tomás G.D., Santos M.S., Moreira P.I., Socorro S., Cavaco J.E., Alves M.G., Oliveira P.F. (2014). Pre-diabetes alters testicular PGC1-α/SIRT3 axis modulating mitochondrial bioenergetics and oxidative stress. Biochim. Biophys. Acta.

[163] Aitken R.J., Harkiss D., Buckingham D.W. (1993). Analysis of lipid peroxidation mechanisms in human spermatozoa. Mol. Reprod. Dev..

[164] Barbonetti A., Cinque B., Vassallo M.R., Mineo S., Francavilla S., Cifone M.G., Francavilla F. (2011). Effect of vaginal probiotic lactobacilli on in vitro-induced sperm lipid peroxidation and its impact on sperm motility and viability. Fertil. Steril..

[165] Wagner B.A., Buettner G.R., Burns C.P. (1994). Free radical-mediated lipid peroxidation in cells: Oxidizability is a function of cell lipid bis-allylic hydrogen content. Biochemistry.

[166] Tremellen K. (2008). Oxidative stress and male infertility--a clinical perspective. Hum. Reprod. Update.

[167] Rato L., Alves M.G., Dias T.R., Lopes G., Cavaco J.E., Socorro S., Oliveira P.F. (2013). High-energy diets may induce a pre-diabetic state altering testicular glycolytic metabolic profile and male reproductive parameters. Andrology.

[168] Wu C.C., Bratton S.B. (2013). Regulation of the intrinsic apoptosis pathway by reactive oxygen species. Antioxid. Redox Signal..

[169] Rato L., Alves M.G., Silva B.M., Sousa M., Oliveira P.F. (2016). Sirtuins: Novel Players in Male Reproductive Health. Curr. Med. Chem..

[170] Palmer N.O., Fullston T., Mitchell M., Setchell B.P., Lane M. (2011). SIRT6 in mouse spermatogenesis is modulated by diet-induced obesity. Reprod. Fertil. Dev..

[171] Metzler-Guillemain C., Depetris D., Luciani J.J., Mignon-Ravix C., Mitchell M.J., Mattei M.G. (2008). In human pachytene spermatocytes, SUMO protein is restricted to the constitutive heterochromatin. Chromosome Res..

[172] Vigodner M., Ishikawa T., Schlegel P.N., Morris P.L. (2006). SUMO-1, human male germ cell development, and the androgen receptor in the testis of men with normal and abnormal spermatogenesis. Am. J. Physiol. Endocrinol. Metab..

[173] Rogers R.S., Inselman A., Handel M.A., Matunis M.J. (2004). SUMO modified proteins localize to the XY body of pachytene spermatocytes. Chromosoma.

[174] Vigodner M. (2009). Sumoylation precedes accumulation of phosphorylated H2AX on sex chromosomes during their meiotic inactivation. Chromosome Res..

[175] Vigodner M., Morris P.L. (2005). Testicular expression of small ubiquitin-related modifier-1 (SUMO-1) supports multiple roles in spermatogenesis: Silencing of sex chromosomes in spermatocytes, spermatid microtubule nucleation, and nuclear reshaping. Dev. Biol..

[176] Seifert E.L., Caron A.Z., Morin K., Coulombe J., He X.H., Jardine K., Dewar-Darch D., Boekelheide K., Harper M.E., McBurney M.W. (2012). SirT1 catalytic activity is required for male fertility and metabolic homeostasis in mice. Fed. Am. Soc. Exp. Biol. J..

[177] Mostafa T., Nabil N., Rashed L., Makeen K., El-Kasas M.A., Mohamaed H.A. (2018). Seminal SIRT1 expression in infertile oligoasthenoteratozoospermic men with varicocoele. Andrology.

[178] Alam F., Syed H., Amjad S., Baig M., Khan T.A., Rehman R. (2021). Interplay between oxidative stress, SIRT1, reproductive and metabolic functions. Curr. Res. Physiol..

[179] Mostafa T., Nabil N., Rashed L., Abo-Sief A.F., Eissa H.H. (2020). Seminal SIRT1-oxidative stress relationship in infertile oligoasthenoteratozoospermic men with varicocele after its surgical repair. Andrologia.

[180] Liu C., Song Z., Wang L., Yu H., Liu W., Shang Y., Xu Z., Zhao H., Gao F., Wen J. (2017). Sirt1 regulates acrosome biogenesis by modulating autophagic flux during spermiogenesis in mice. Development.

[181] Di Emidio G., Falone S., Artini P.G., Amicarelli F., D’Alessandro A.M., Tatone C. (2021). Mitochondrial Sirtuins in Reproduction. Antioxidants.

[182] Ahn B.H., Kim H.S., Song S., Lee I.H., Liu J., Vassilopoulos A., Deng C.X., Finkel T. (2008). A role for the mitochondrial deacetylase Sirt3 in regulating energy homeostasis. Proc. Natl. Acad. Sci. USA.

[183] Vertika S., Singh K.K., Rajender S. (2020). Mitochondria, spermatogenesis, and male infertility—An update. Mitochondrion.

[184] Barbagallo F., La Vignera S., Cannarella R., Aversa A., Calogero A.E., Condorelli R.A. (2020). Evaluation of Sperm Mitochondrial Function: A Key Organelle for Sperm Motility. J. Clin. Med..

[185] Bello J.H., Khan M.J., Amir S., Kakakhel H.G., Tahir F., Sultan S., Raza S.Q., Mamoulakis C., Zachariou A., Tsatsakis A. (2022). Dysregulation of mitochondrial sirtuin genes is associated with human male infertility. Andrologia.

[186] North B.J., Marshall B.L., Borra M.T., Denu J.M., Verdin E. (2003). The human Sir2 ortholog, SIRT2, is an NAD^+^-dependent tubulin deacetylase. Mol. Cell.

[187] Loganathan C., Kannan A., Panneerselvam A., Mariajoseph-Antony L.F., Kumar S.A., Anbarasu K., Prahalathan C. (2021). The possible role of sirtuins in male reproduction. Mol. Cell. Biochem..

[188] Mao Z., Hine C., Tian X., Van Meter M., Au M., Vaidya A., Seluanov A., Gorbunova V. (2011). SIRT6 promotes DNA repair under stress by activating PARP1. Science.

[189] Wei H., Khawar M.B., Tang W., Wang L., Wang L., Liu C., Jiang H., Li W. (2020). Sirt6 is required for spermatogenesis in mice. Aging.

[190] Vazquez B.N., Thackray J.K., Simonet N.G., Kane-Goldsmith N., Martinez-Redondo P., Nguyen T., Bunting S., Vaquero A., Tischfield J.A., Serrano L. (2016). SIRT7 promotes genome integrity and modulates non-homologous end joining DNA repair. Eur. Mol. Biol. Organ. J..

